# Nuclear Receptor-Mediated Alleviation of Alcoholic Fatty Liver by Polyphenols Contained in Alcoholic Beverages

**DOI:** 10.1371/journal.pone.0087142

**Published:** 2014-02-03

**Authors:** Ruiqing Yao, Akihito Yasuoka, Asuka Kamei, Shota Ushiama, Yoshinori Kitagawa, Tomohiro Rogi, Hiroshi Shibata, Keiko Abe, Takumi Misaka

**Affiliations:** 1 Department of Applied Biological Chemistry, Graduate School of Agricultural and Life Sciences, The University of Tokyo, Bunkyo-ku, Tokyo, Japan; 2 Department of Biological Engineering, Maebashi Institute of Technology, Maebashi-shi, Gunma, Japan; 3 Kanagawa Academy of Science and Technology, Takatsu-ku, Kawasaki-shi, Kanagawa, Japan; 4 Institute for Health Care Science, Suntory Wellness Ltd., Shimamoto-cho, Mishima-gun, Osaka, Japan; Beckman Research Institute of City of Hope, United States of America

## Abstract

To elucidate the effect of the polyphenols contained in alcoholic beverages on the metabolic stress induced by ethanol consumption, four groups of mice were fed for five weeks on Lieber's diet with or without ethanol, with ethanol plus ellagic acid, and with ethanol plus trans-resveratrol. Alcoholic fatty liver was observed in the group fed the ethanol diet but not in those fed the ethanol plus polyphenol diets. Liver transcriptome analysis revealed that the addition of the polyphenols suppressed the expression of the genes related to cell stress that were up-regulated by ethanol alone. Conversely, the polyphenols up-regulated the genes involved in bile acid synthesis, unsaturated fatty acid elongation, and tetrahydrofolate synthesis that were down-regulated by ethanol alone. Because parts of these genes were known to be regulated by the constitutive androstane receptor (CAR), we performed the same experiment in the CAR-deficient mice. As a result, fatty liver was observed not only in the ethanol group but also with the ethanol plus polyphenol groups. In addition, there was no segregation of the gene expression profiles among these groups. These results provide a molecular basis for the prevention of alcohol-induced stress by the polyphenols in alcoholic beverages.

## Introduction

Alcoholic beverages contain a certain number of phytochemicals, most of which contain aromatic rings [1], [2]. For example, red wines contain various amounts of trans-resveratrol (RSV), a stilbene derived from grape peel. Grape peel also includes flavones and anthocyanidins [3]. Ellagic acid (EA) is generated from wood tannins during whisky aging in a barrel [4]. Beer contains several chalcones derived from hops [1], [2]. Whereas ethanol, which is the major components of alcoholic beverages, undergoes metabolic processes that have several effects on energy homeostasis, as described below, these phytochemicals have been known to exhibit various medicative effects on metabolic abnormalities. RSV is famous for the causative factor of French paradox and has a protective effect against cardiovascular diseases [3], [5], [6]. EA is reported to inhibit the inflammation of vascular tissues [5], [7], [8]. Hops chalcones have the ability to decrease blood lipid levels [5], [9], [10]. Parts of these effects are attributable to their antioxidative activities because reactive oxygen species (ROS) are thought to play the main causative roles in these metabolic abnormalities [11]–[13]. While the bioavailability and the tissue distribution of these phytochemicals vary depending on their molecular nature, they are thought to act on the ROS sources over a broad spectrum. However, there is emerging evidence that these phytochemicals interact with molecular targets to exert ameliorative effects on metabolic syndromes.

RSV has been reported to influence the activities of the key metabolic regulators, such as NAD-dependent protein deacetylases (SIRTs) and adenosine monophosphate activated kinase (AMPK). These molecules function as sensors for lowered energy status (increase in NAD and AMP concentration, respectively), and the action of RSV is considered to be mimetic of calorie restriction [3], [14]. There has been disagreement about the direct target of RSV, but recent research has raised the possibility that phosphodiesterases (PDEs) may be the farthest upstream target in the AMPK - SIRT signaling axis [15]. RSV is also reported to activate the other metabolic regulators, the nuclear receptors (NRs). Peroxisome proliferator-activated receptor (PPAR: NR1C1, 2, and 3) is one of the NRs of this kind, and it receives fatty acid derivatives as endogenous ligands to regulate lipid homeostasis [16], [17]. Because the synthetic PPAR agonists, which have been used as drugs for type 2 diabetes, cause liver damage, food-derived phytochemicals showing PPAR agonistic activity are attracting attention from the standpoint of disease prevention [18]–[20]. The constitutive androstane receptor (CAR: NR1I3), which belongs to the NR1I subfamily, and its relative, the pregnene X receptor (PXR: NR1I2), is the other one that responds to phytochemicals [21]. CAR is known as a drug-responsive nuclear receptor that induces the detoxification of xenobiotics, but recent studies have revealed its expanded roles in regulating the genes related to energy metabolism [22]. Two groups have reported on the amelioration of fatty liver symptom by the artificial mouse CAR activator, 1,4-Bis(3,5-dichloro-2-pyridyloxy)benzene (TCPOBOP), one in ob/ob mice and the other in mice fed high fat diet [59], [60]. But this chlorinated phenolic compound has hepatocarcinogenic effect, and thus can not be a suitable model for food mediated therapy of fatty liver. We have screened 32 phytochemicals for their capacity to activate CAR and found that some hydroxyl flavones, including chrysin (5, 7-OH flavone), RSV, and EA, elicited the activities as strongly as known artificial CAR activators. In particular, chrysin induced *cyp2b10* gene expression in mouse liver in a CAR-dependent manner, suggesting the possibility that food-derived polyphenols can regulate CAR, alleviating metabolic abnormalities [23], [24].

Under the condition of appropriate energy provision, chronic alcohol intake inflicts progressive damage on energy metabolism, causing alcoholic fatty liver (AFL) and insulin resistance, finally leading to the diseases such as cirrhosis and diabetes [11], [12], [25], [26]. Therefore, prevention of AFL by food factors, not by drugs, provides good health benefits. Absorbed ethanol undergoes oxidation steps catalyzed by three types of enzymes—cytosolic alcohol dehydrogenase (ADH), microsomal CYP2E and peroxisomal catalase—producing acetaldehyde, which is further oxidized to acetic acid by mitochondrial acetaldehyde dehydrogenase (ALDH). These enzymatic systems have been implicated as the sources of ROS, and parts of them (those by ADH and ALDH) are coupled with the reduction of nicotinamide adenine dinucleotide (NAD) to NADH. It has been shown that ROS inactivate the enzymes in β-oxidation and the TCA cycle [12], [25]. In addition, the excess supply of NADH inhibits the NADH-producing steps of these catabolic pathways [12], [25]. These impacts on the metabolic pathways are thought to trigger an out-of-balance state of lipid metabolism within liver and to promote AFL.

Transcriptomic analyses of AFL progression have been conducted by several groups; the researchers generally focused on the monophasic changes of gene expression in parallel with aggravation of the symptoms [27]–[29]. One of the weaknesses of this type of approach is that the way to select genes basically depends on changes in their expression levels, provided that there is no supporting physiological data about such genes. This can lead to misidentification of genes of less importance or overlooking potentially relevant genes. Additionally, it should be noted that this type of study tends to highlight genes involved in the worsening of liver diseases, not in the amelioration of it. In this study, we have examined the effects of two types of polyphenols, EA and RSV, on the progression of AFL in a mouse model. Because they showed similar suppressive effects on AFL, we conducted a liver transcriptome analysis, tracing the biphasic change of the symptoms, *i.e.*, from the normal state to fat accumulation and amelioration, to extract genes that would help to induce amelioration.

## Materials and Methods

### Mouse maintenance

All animals were maintained following the Guide for the care and use of laboratory animals provided by the Ministry of Education, Culture, Sports, Science, and Technology, Japan. Five-week-old C3H/HeN female mice (CLEA, Japan) were acclimated to the maintenance condition (25°C, 8:00–20:00 day/20:00–8:00 night cycle and 35∼40% humidity), fed a CE-2 diet (CLEA, Japan), and given water *ad libitum* for one week. Each group of mice (n = 4 for wild type mice analysis and n = 3 for CAR decficient mice analysis) was fed Lieber's isocaloric diet (Oriental yeast, Japan) containing water, containing ethanol, containing ethanol and ellagic acid (Fluka Biochemika, Switzerland), or containing ethanol and *trans*-resveratrol (Sigma, USA) (Table S1) for one week at 10:00 *ad libitum*. Then, the mice were fed each diet at 12 g/day for four weeks (Fig. S1A). The approximate intake of each polyphenol was 50 mg/kg body weight/day. At 10:00 of the final day of the experimental period, the animals were anesthetized by diethyl ether, sacrificed by cervial fracture, and the heart blood, and the liver were collected. The serum biochemical analyses were conducted by Nagahama Science Laboratory (Japan). The protocol for the animal experiments was approved by the Animal Use Committee of the Faculty of Agriculture at The University of Tokyo. CAR-dificient (C3H/HeN background) mouse strain was kindly provided by Dr. Negishi Masahiko in National Institute of Environmental Health Sciences, National Institute of Health, USA.

### Histochemical analysis

The right and left lobes of the liver in each mouse were dissected and stored at −80°C. Each part was cryosectioned (10 micro-m), air dried, fixed in phosphate-buffered saline containing 4% formaldehyde for 10 min, and rinsed in 60% isopropanol. Sections were stained in 60% isopropanol containing 0.3% Oil Red-O (Sigma, USA) for 15 min, rinsed in 60% isopropanol, and mounted. Sections were observed under a microscope (IX70, Olympus, Tokyo, Japan), and the images of each section were obtained using a DP70 digital camera system (Olympus, Tokyo, Japan). The staining intensity (SI) of Oil Red-O was determined by integrating the pixel signal levels on the red color channel using Photoshop® (Adobe Systems, San Jose, CA) and Image J free software. Then, the SI value of the red color channel was divided by that of the blue color channel to obtain the normalized SI value. The normalized SI values of the right and left lobe sections (two images for each lobe section) were averaged and used as the SI value of each mouse.

### DNA microarray analysis

Total RNA was isolated from 50 mg of mouse liver using ISOGEN (Nippon gene, Japan) and purified using an RNeasy Mini Kit (Qiagen, USA). The quality of the purified RNA was examined by the Agilent RNA 6000 Nano Assay protocol (Agilent technologies, Japan) and reverse-transcribed into cDNA using a One-Cycle cDNA Synthesis Kit (Affymetrix, Japan). A biotin-labeled anti-sense RNA probe was synthesized from cDNA using a GeneChip® 3′ IVT Express Kit (Affymetrix, Japan), fragmented, and hybridized with GeneChip® Mouse Genome 430 2.0 Array (Affymetrix, Japan) for 16 hr. The chip was washed, stained, and scanned with Affymetrix GeneChip Command Console software (Affymetrix, Japan). The probe signals were normalized by the Distribution Free Weighted (DFW) method, the Affymetrix Microarray Suite version 5.0 (MAS), the Factor Analysis for Robust Microarray Summarization (qFARMS), or the Robust Multi-array Averaging (RMA) on the R 2.7 or the R 2.12. platform, and the probes were selected according to the false discovery rate (FDR) values obtained by comparison of expression levels among the experimental groups. The selected gene sets were analyzed by the Database for Annotation, Visualization, and Integrated Discovery (DAVID; http//david.abcc.ncifcrf.gov/) [30] for their significant representation in certain Gene Ontology (GO) terms. All microarray data were submitted to The National Center for Biotechnology Information Gene Expression Ominibus section (Accession number: GSE52644).

## Results

### Alleviation of alcoholic fatty liver by ellagic acid and trans-resveratrol

**Figure 1:**
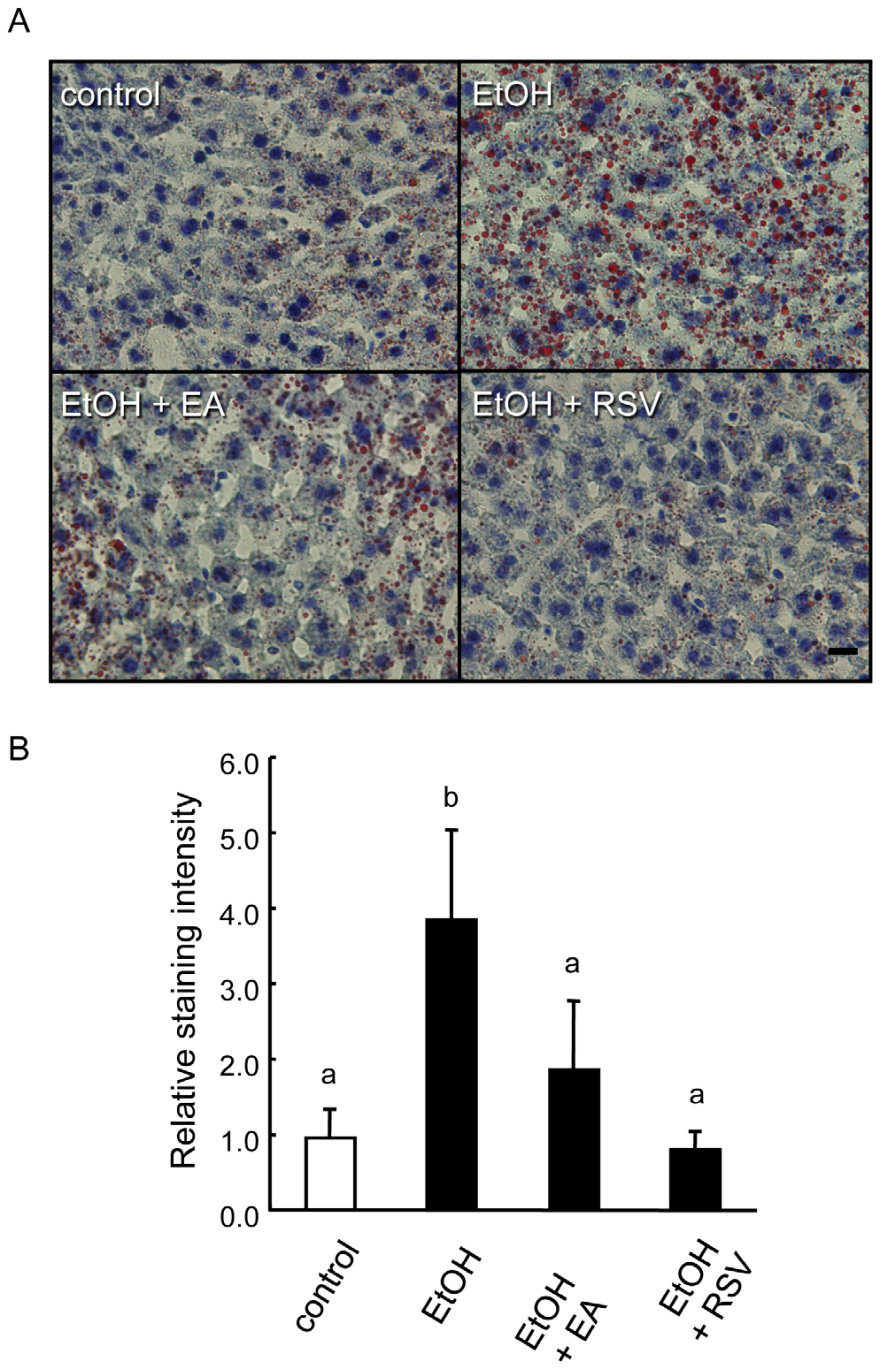
Effect of polyphenols on the alcohol-induced fatty liver in the mice. A, Oil red O staining of the liver sections from the mice fed the control, the ethanol, the ethanol plus ellagic acid, or the ethanol plus trans-resveratrol diet. A larger number of oil drops was observed in the section of the mouse fed the ethanol diet compared with the other sections. B, Quantification of the oil drop intensities per section by image analysis (see Materials and Methods). Differences (n = 4 and 5, Tukey-Kramer multiple comparison) were observed between the ethanol group (b) and the other groups (a). Scale bar, 10 µm.

Mice were fed one of four liquid diets for five weeks (Table S1, Fig. S1A) and sacrificed for observation of the fat accumulation in the liver. There was no difference in body weight or in relative liver weight among the treated groups (Fig. S1B and C). However, densely stained oil droplets were observed in the mice fed the EtOH diet (Fig. 1A). The quantification of staining intensities revealed that this accumulation was four times higher than the accumulation in mice fed control diet (Fig. 1B, control and EtOH). In contrast, the addition of EA or RSV to the diet formula significantly reduced this steatosis close to the control level (Fig. 1B). Analysis of the blood samples of each animal group revealed that triacylglycerol levels were reduced in all groups fed alcohol-containing diets and High-density lipoprotein (HDL) cholesterol levels were increased only in the groups fed the polyphenols (marked with “b”, Table S2). No significant between-group difference was observed in the concentrations of liver damage markers such as glutamic-pyruvic transaminase and glutamic-oxaloacetic transaminase (not shown).

### Extraction of genes correlated with the alleviation of alcoholic fatty liver

**Figure 2:**
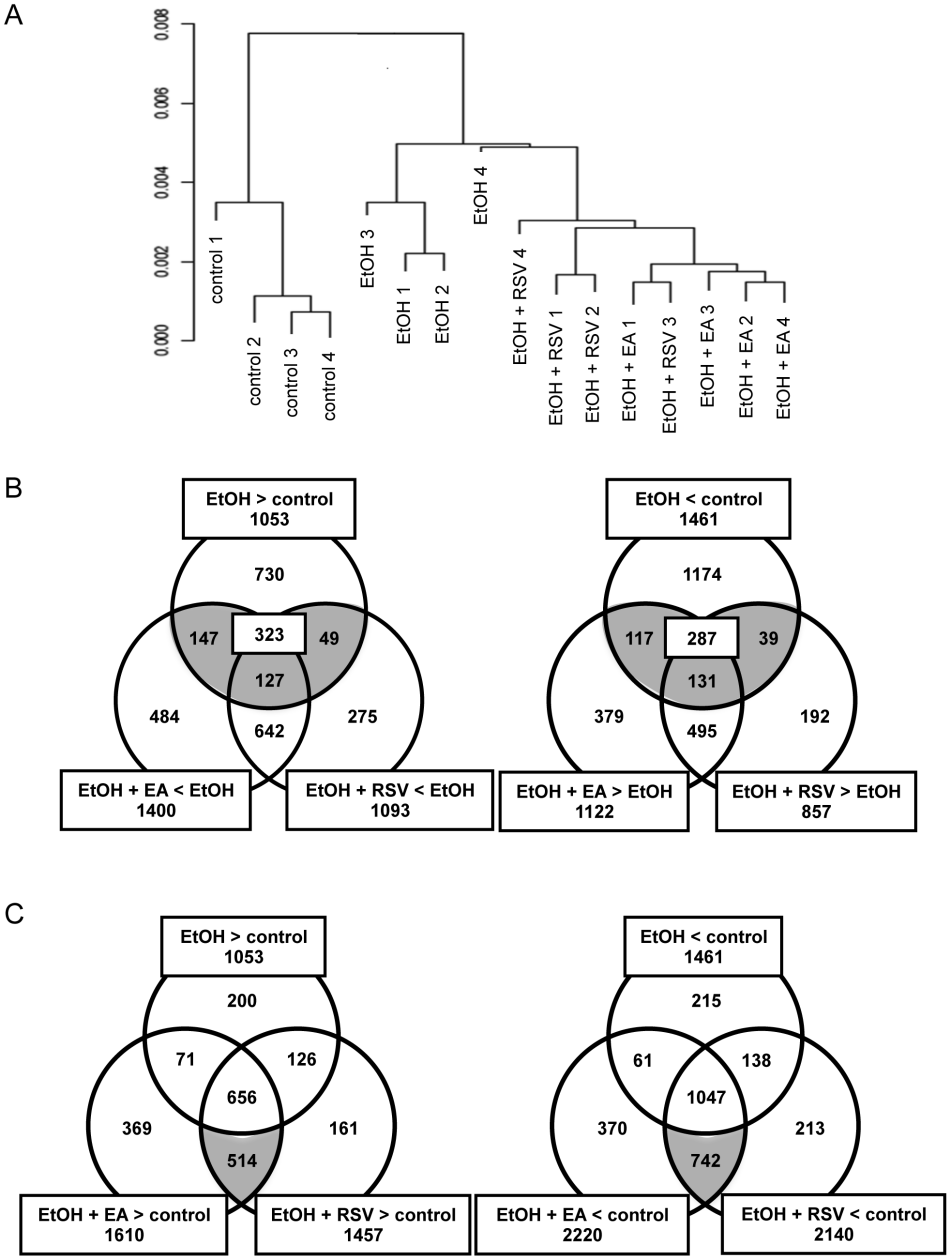
DNA microarray analysis of the liver mRNA from the mice fed the various diets. A, Cluster analysis of the gene expression profiles. Gene expression levels were normalized by the qFARMs method. The numbers at the end of the fed condition represent the mouse individuals. All the bootstrap values were larger than 70 (not shown). B, Numbers of the genes whose expression levels were affected by each feeding condition. Genes were selected based on their FDR values (<0.05) calculated by multiple comparisons of expression levels (n = 4) among feeding conditions (Et, ethanol; con, control; Ella, ethanol plus ellagic acid; Res, ethanol plus trans-resveratrol), e.g., up-regulated in the ethanol group compared with the control group (Et>con). Genes were then categorized in reference to their multiplicity of occurrence in each set, as indicated by the Benn diagram.

To unravel the molecular mechanisms underlying these alleviative effects of the polyphenols, we compared the liver gene expression profiles of the groups using DNA microarray analysis. When the gene expression levels were normalized by the qFARMS method, better segregation of the clusters that correspond to the diet groupings was observed (Fig. 2A). The probe sets were then selected according to their FDR values (>0.025), calculated from a comparison among the groups by the rank product method [31]. Next, we used two different strategies to extract genes whose regulation correlates with the amelioration of steatosis. One strategy is to select genes that are differentially regulated by the ethanol plus polyphenol diets compared with the ethanol diet (Fig. 2B). The other strategy is to select those genes regulated only by the ethanol plus polyphenol diets (Fig. 2C). We detected a 1053 probe set that was up-regulated by the ethanol diet compared with the control diet (Fig. 2B, left). Among these, a 147+127+49 ( = 323) probe set was oppositely (down-) regulated in the ethanol plus EA group or in the ethanol plus RSV group. A similar analysis of the probe sets that were down-regulated by the ethanol treatment (the 1461 probe set) resulted in the identification of a 117+131+39 ( = 287) probe set that was oppositely (up-) regulated in the ethanol plus polyphenol groups (Fig. 2B, right). Next, we compared the 1053 probe set with those up-regulated by the ethanol plus polyphenol diets and found that the 514 probe set was specific to the latter diets (Fig. 2C, left). For the 1461 genes, the 742 probe set was found to be down-regulated only by the ethanol plus polyphenol diets (Fig. 2C, right). These probe sets were then subjected to a gene ontology analysis using the Biological Process database [30]. Tables 1A, 2A, 3A, and 4A represent the functional groupings in which the 323, 287, 514, and 742 probe sets showed a significant extent of enrichment (p<0.05), respectively. We then focused on the terms located at the lowest levels of the category hierarchies.

**Table 1:** Enrichment analysis of 323 probe set whose expression levels were up-regulated by ethanol and down-regulated by the addition of polyphenols.

GO-terms	p value
GO:0010033∼response to organic substance	1.75E-02
GO:0051789∼response to protein stimulus	1.60E-03
GO:0006986∼response to unfolded protein*	9.22E-04
GO:0051603∼proteolysis involved in cellular protein catabolic process	2.24E-02
GO:0010498∼proteasomal protein catabolic process	9.36E-03
GO:0043161∼proteasomal ubiquitin-dependent protein catabolic process*	9.36E-03
GO:0031016∼pancreas development*	1.57E-02
GO:0051246∼regulation of protein metabolic process*	1.34E-02
GO:0006458∼‘de novo’ protein folding	1.84E-02
GO:0051084∼‘de novo’ posttranslational protein folding	1.84E-02
GO:0051085∼chaperone mediated protein folding requiring cofactor*	1.36E-02
GO:0005996∼monosaccharide metabolic process	2.09E-02
GO:0019318∼hexose metabolic process	1.17E-02
GO:0006006∼glucose metabolic process*	1.57E-02
GO:0043603∼cellular amide metabolic process*	2.10E-02
GO:0031324∼negative regulation of cellular metabolic process*	1.37E-02
GO:0046907∼intracellular transport	1.29E-02
GO:0006886∼intracellular protein transport*	2.33E-02
GO:0030512∼negative regulation of TGF beta receptor signaling pathway*	2.38E-02

* : Lowest level groupings in which significant enrichment was observed.

**Table 2:** Enrichment analysis of 287 probe set whose expression levels were down-regulated by ethanol and up-regulated by the addition of polyphenols.

GO-terms	p value
GO:0055114∼oxidation reduction*	7.13E-03
GO:0006629∼lipid metabolic process	5.75E-05
GO:0008610∼lipid biosynthetic process	1.65E-03
GO:0006694∼steroid biosynthetic process*	4.41E-04
GO:0006066∼alcohol metabolic process	2.44E-02
GO:0008202∼steroid metabolic process	8.17E-05
GO:0016125∼sterol metabolic process	4.29E-03
GO:0008203∼cholesterol metabolic process*	1.61E-02
GO:0044255∼cellular lipid metabolic process	1.31E-02
GO:0006631∼fatty acid metabolic process	1.12E-02
GO:0006633∼fatty acid biosynthetic process*	2.21E-02
GO:0006869∼lipid transport*	2.09E-02
GO:0055085∼transmembrane transport*	2.15E-02

* : Lowest level groupings in which significant enrichment was observed.

**Table 3:** Enrichment analysis of 514 probe set whose expression levels were up-regulated only in the presence of ethanol plus polyphenols.

GO-terms	p value
GO:0007623∼circadian rhythm*	1.11E-02
GO:0006629∼lipid metabolic process	2.50E-04
GO:0008202∼steroid metabolic process*	2.24E-02
GO:0006807∼nitrogen compound metabolic process	7.50E-04
GO:0034641∼cellular nitrogen compound metabolic process	4.91E-03
GO:0043603∼cellular amide metabolic process*	1.77E-02
GO:0044271∼nitrogen compound biosynthetic process*	2.10E-02
GO:0042364∼water-soluble vitamin biosynthetic process*	2.07E-02
GO:0051186∼cofactor metabolic process	2.03E-04
GO:0006732∼coenzyme metabolic process	1.32E-05
GO:0009108∼coenzyme biosynthetic process*	2.47E-02
GO:0005976∼polysaccharide metabolic process	1.82E-03
GO:0006022∼aminoglycan metabolic process	N/A
GO:0030203∼glycosaminoglycan metabolic process*	1.48E-02
GO:0006760∼folic acid and derivative metabolic process	4.04E-03
GO:0046653∼tetrahydrofolate metabolic process*	6.34E-03
GO:0019752∼carboxylic acid metabolic process	1.00E-08
GO:0032787∼monocarboxylic acid metabolic process	1.10E-05
GO:0006631∼fatty acid metabolic process*	5.35E-03
GO:0009109∼coenzyme catabolic process	6.26E-03
GO:0046356∼acetyl-CoA catabolic process	2.32E-02
GO:0006099∼tricarboxylic acid cycle*	2.07E-02
GO:0006446∼regulation of translational initiation*	1.61E-02
GO:0006520∼cellular amino acid metabolic process	3.45E-03
GO:0000096∼sulfur amino acid metabolic process	1.53E-03
GO:0006555∼methionine metabolic process*	2.13E-02
GO:0042278∼purine nucleoside metabolic process	2.32E-02
GO:0009119∼ribonucleoside metabolic process	1.77E-02
GO:0046128∼purine ribonucleoside metabolic process	2.32E-02
GO:0046500∼S-adenosylmethionine metabolic process*	9.34E-03
GO:0006006∼glucose metabolic process	2.37E-03
GO:0010906∼regulation of glucose metabolic process	4.04E-03
GO:0006111∼regulation of gluconeogenesis*	9.34E-03
GO:0046218∼indolalkylamine catabolic process	6.34E-03
GO:0006569∼tryptophan catabolic process	6.34E-03
GO:0019441∼tryptophan catabolic process to kynurenine*	3.87E-03

* : Lowest level groupings in which significant enrichment was observed.

**Table 4:** Enrichment analysis of 742 probe set whose expression levels were down-regulated only in the presence of ethanol plus polyphenols.

GO-terms	p value
GO:0044087∼regulation of cellular component biogenesis*	1.75E-02
GO:0044267∼cellular protein metabolic process	1.04E-06
GO:0006464∼protein modification process	N/A
GO:0006457∼protein folding	1.75E-02
GO:0043687∼post-translational protein modification*	1.05E-02
GO:0006467∼protein thiol-disulfide exchange*	8.04E-03
GO:0006412∼translation	1.66E-05
GO:0006413∼translational initiation*	9.39E-03
GO:0034641∼cellular nitrogen compound metabolic process	3.13E-05
GO:0044106∼cellular amine metabolic process	5.87E-03
GO:0006520∼cellular amino acid metabolic process*	1.10E-02
GO:0048008∼platelet-derived growth factor receptor signaling pathway*	7.09E-03
GO:0008213∼protein amino acid alkylation	1.81E-02
GO:0006479∼protein amino acid methylation*	1.81E-02
GO:0007431∼salivary gland development	2.49E-02
GO:0007435∼salivary gland morphogenesis*	1.74E-02
GO:0006461∼protein complex assembly	4.13E-03
GO:0043623∼cellular protein complex assembly	N/A
GO:0051258∼protein polymerization*	1.33E-02
GO:0070647∼protein modification by small protein conjugation or removal	7.34E-05
GO:0032446∼protein modification by small protein conjugation	5.85E-04
GO:0016567∼protein ubiquitination*	2.89E-03
GO:0016070∼RNA metabolic process	2.31E-05
GO:0006396∼RNA processing	3.08E-05
GO:0008380∼RNA splicing*	5.60E-05
GO:0006397∼mRNA processing*	4.42E-07
GO:0045934∼negative regulation of nucleobase, nucleoside, nucleotide and nucleic acid metabolic process	2.09E-02
GO:0016481∼negative regulation of transcription*	1.94E-02
GO:0006829∼zinc ion transport*	8.41E-03
GO:0019941∼modification-dependent protein catabolic process	5.39E-06
GO:0006511∼ubiquitin-dependent protein catabolic process	9.87E-08
GO:0043161∼proteasomal ubiquitin-dependent protein catabolic process*	6.20E-04
GO:0016568∼chromatin modification	1.15E-03
GO:0016569∼covalent chromatin modification	2.36E-02
GO:0016570∼histone modification*	1.86E-02
GO:0006357∼regulation of transcription from RNA polymerase II promoter*	6.75E-03

* : Lowest level groupings in which significant enrichment was observed.

### Characteristics of the probe sets whose expression levels were modulated by the addition of the polyphenols to the diets

A total of 323 probe sets showed significant enrichment in 10 GO-terms (GO-terms) located at the lowest levels of the category hierarchy (Table 1). Five of these were related to protein folding and degradation (GO:0006986, GO:0043161, GO:0051246, GO:0051085, and GO:0006886). Three categories seemed to be involved in carbohydrate metabolism (GO:0006006, GO:0043603, and GO:0031324). This indicates that the polyphenols down-regulate genes related to protein folding stress and carbohydrate metabolism, which were once up-regulated by feeding with ethanol alone. The other 2 categories (GO:0031016 and GO:0030512) seemed to be involved in organ developmental processes. In the case of the 287 probe set, different types of GO-terms (six terms) were observed compared with those for the 323 probe set (Table 2). Most of the categories were related to lipid homeostasis (GO:0055114, GO:0006694, GO:0008203, GO:0006633, and GO:0006869) except for the one (GO:0055085) related to transmembrane transport, suggesting that the polyphenols up-regulate genes mainly related to lipid metabolism, which were once down-regulated by ethanol alone. The 514 probe set showed a higher number of GO-terms (15 terms, Table 3) than the 323 and 287 probe sets did. One of the notable characteristics is the occurrence of three terms related to one-carbon metabolism (GO:0046653, GO:0006555, and GO:0046500). A term for circadian rhythm (GO:0007623) is also unique to this gene group. Two terms were related to lipid metabolism (GO:0008202 and GO:0006631), of which subcategories are also observed with 287 gene sets (Table 1). Seven categories seemed to be involved in energy production: nicotine amide production (GO:0042364, GO:0043603, and GO:0019441), TCA cycle (GO:0006099), gluconeogenesis (GO:0006111), and vitamin synthesis (GO:0044271 and GO:0009108). Other categories included the glycosaminoglycan metabolic process (GO:0030203) and regulation of translational initiation (GO:0006446). In contrast to the other probe sets, the 742 probe set exhibited enrichment in a large number of terms (17 terms) at the lowest levels (Table 4). Five of these (GO:0043687, GO:0006467, GO:0051258, GO:0016567, and GO:0043161) controlled protein folding and degradation processes, showing similarity to the case of the 323 probe set. Four terms (GO:0008380, GO:0006397, GO:0016481, and GO:0006357) were identified as relevant to mRNA synthesis. Two categories (GO:0006479 and GO:0016570) seemed to be related to chromatin modification. Half of the rest of the terms (GO:0044087, GO:0006413, and GO:0006520) showed relatively general cellular functions, and the other terms (GO:0048008, GO:0007435, and GO:0006829) may be involved in specific functions such as development and zinc ion transport. However, all of these terms ranked so highly in the category hierarchy that it was difficult to identify any specific function among these terms. Therefore, we omitted this probe set from the objectives of the following analysis.

### Functional annotation of the extracted genes

**Figure 3:**
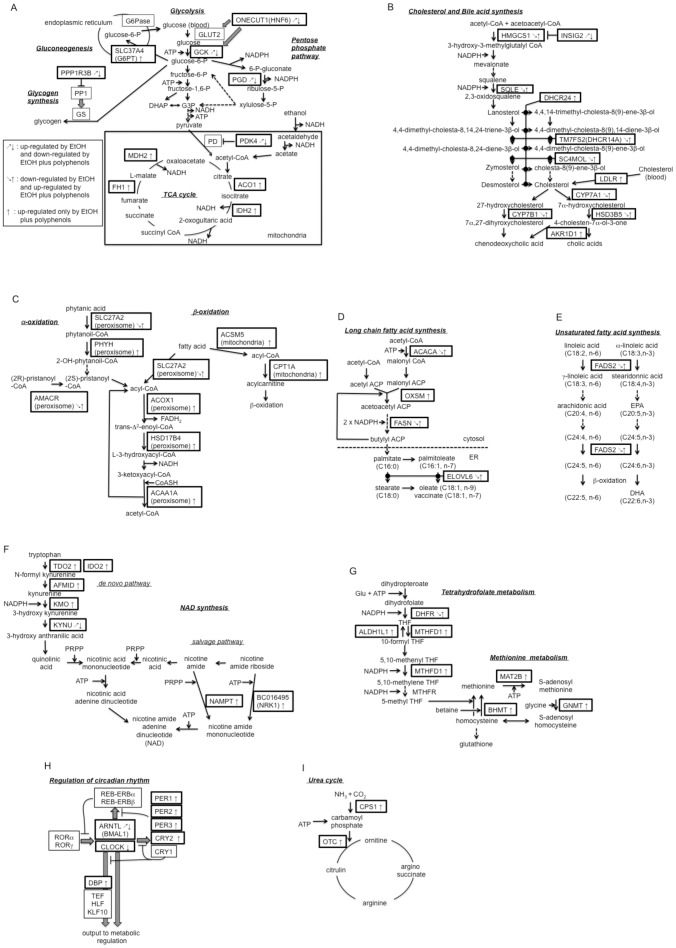
Mapping of the extracted gene products in metabolic pathways.

Of the 323 probe set, 52 genes showed significant enrichment in the 10 categories (Table S3). We paid particular attention to the genes related to carbohydrate and lipid metabolism because fat accumulation induced by alcohol intake accompanies the malfunctioning of these metabolism processes [25]. We found two enzymes—glucokinase (GCK) and phosphogluconate dehydrogenase (PGD)—and the three regulatory proteins—protein phosphatase 1 regulatory subunit 3b (PPP1R3B), one cut domain family member 1 (ONECUT1), and pyruvate dehydrogenase kinase 4 (PDK4)—among the gene products attributed to the GO:0006006 glucose metabolic process. These gene products were mapped in the metabolic pathways, as illustrated in Fig. 3A. The gene products that are involved in the TCA cycle (ACO1, IDH2, FH1, and MDH2) will be described in a later section. Most of the other gene products in Table 1B seemed to play roles in protein folding processes, as predicted from their attributed GO-terms. As for the 287 probe set, 41 genes showed significant enrichment in six GO-terms, of which five terms were related to lipid metabolism (Table S4). There were 10 genes—*Cyp7a1*, *Cyp7b1*, *Fads2*, *Fasn*, *Hmgcs1*, *Hsd3b5*, *Sc4mol*, *Sdr42e1*, *Spns2*, and *Tm7fs2* (*Dhcr14a*)—appearing multiple times in these GO categories. Analyses of each gene function revealed that seven gene products (CYP7A1, CYP7B1, HMGCS1, HSD3B5, SC4MOL, SQLE, and TM7FS2) were engaged in the synthesis of steroids and bile acid (Fig. 3B). This metabolic pathway also includes two enzymes, DHCR24, catalyzing 24-double bond reduction, and AKR1D1, catalyzing 5-beta reduction, as members of 67 gene products (Table S5). Another characteristic of this gene set was the presence of four gene products (ACACA, FASN, ELOVL6, and FADS2) involved in long-chain fatty acid synthesis and desaturation (Figs. 3D and E). The former metabolic pathway also includes OXSM, constituting 67 gene products. Several genes related to lipid transport were identified (GO:0006869). These gene products were APOL7a, OSBPL3(ORP3), PTPNC1(RDGB), PLTP, SLC27A2(FATP2), and SPNS2, among which SLC27A2 (FATP2) has been reported to function as both a fatty acid transporter and a very long-chain acyl-CoA synthetase in peroxisomes (Fig. 3C) [32]. There were 13 gene products classified into the transmembrane transport category (GO:0055085), but each member showed a diverse function in terms of its substrate; for example, SLC16A2 (MCT8) and SLC16A10 (MCT10) for thyroid [33], SLC22A7 (OAT2) for uric acid [34], and SLC23A1 for vitamin C [35]. A total of 514 gene sets showed a relatively high number of GO-terms (15 terms) among the other gene sets. Out of 67 genes showing significant enrichment in the categories, 11 genes appeared multiple times in the list (Table S5). Five of the 11 genes (*Gch1*, *Mthfd1*, *Bhmt*, *Mat2b*, and *Gnmt*) are involved in one-carbon metabolism (Fig. 3G). This metabolic pathway also includes ALDH1L1 as the member of 67 gene products and DHFR as the member of 41 gene products (Table S4). The other three members of the 11 genes (*Kmo*, *Nampt*, and *Nrk1*) were found to be necessary for NAD synthesis (Fig. 3F). This pathway receives tryptophan as the substrate for *de novo* NAD synthesis. TDO2, IDO2, and AFMID, which were also identified as members of 67 gene products (Table S5), catalyze oxidation-hydrolysis steps of this stream. In addition, KYNU, located at one of these steps, was identified as a member of 52 gene products (Table 1B). The rest of the 11 genes included *Otc* and *Cps*, whose products constitute the urea cycle (Fig. 3I). Another characteristic of the 514 probe set was the enrichment of genes in the categories related to the processes producing energy from sugar or lipid. We found four gene products—MDH2, FH1, ACO1, and IDH2—involved in the TCA cycle (Fig. 3A). Two of them, IDH2 and MDH2, are located at NADH-producing steps in the cycle. As for lipid metabolism, six enzyme genes—*Acaa1a*, *Acsm5*, *Acox1*, *Cpt1a*, *Hsd17b4*, and *Phyh*—were found to be involved in fatty acid oxidation (Fig. 3C). Notably, four of these gene products (ACAA1A, ACOX1, HSD17B4, and PHYH) have been reported to be localized to peroxisome [36] and that the two peroxisomal enzymes, SLC27A2 (FATP2) and AMACR, were also identified as members of the 41 gene products (Table S4) [32], [37]. The most distinct category attributed to the 514 probe set was GO:0007623-circadian rhythm, which contains four genes (*Per1*, *Per2*, *Per3*, and *Cry1*) essential for the core clock oscillation and one gene (*Dbp*) essential for its output to metabolic regulation (Fig. 3H). Other components of the core clock oscillator were also identified in the 52 gene products (*Arntl*, Table S3) and in the 253 gene products (*Clock*, Table S6).

### Dependency of the polyphenols' ameliorative effect on CAR activity

**Figure 4:**
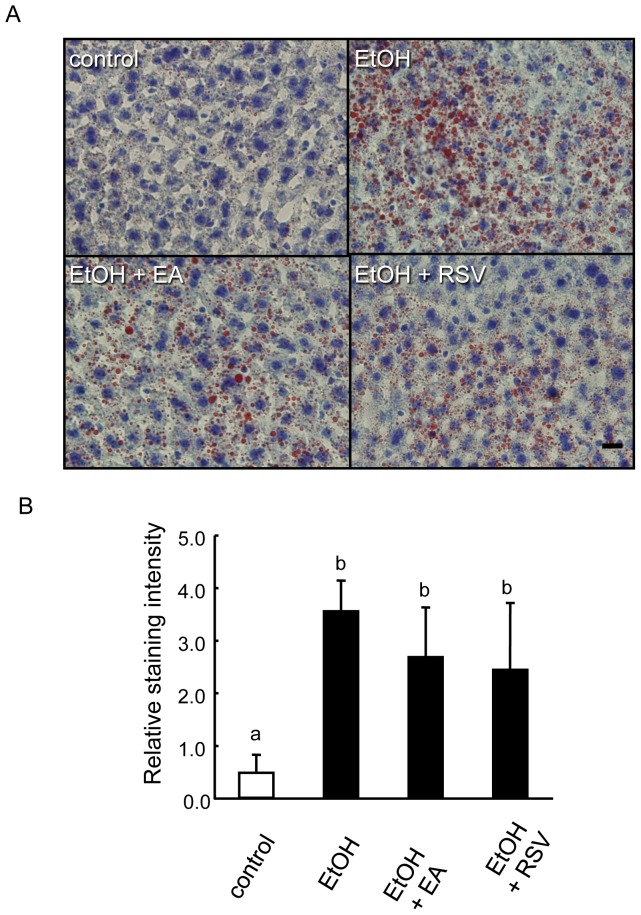
Dependency of the polyphenol's ameliorative effect on the CAR gene. A, Oil red O staining of the liver sections from CAR KO mice fed under the same conditions as described in Fig. 1. Larger numbers of oil drops were observed in the sections of mice fed the ethanol, the ethanol plus ellagic acid, or the ethanol plus trans-resveratrol diet compared with that of the control diet. B, Quantification of oil drop intensities per section. Differences (n = 3, Tukey-Kramer multiple comparison) were observed between the control group (a) and the other groups (b). Scale bar, 10 µm.

Analyzing the extracted genes, we found multiple genes that have been reported to be regulated by CAR; three genes (*Pgd*, *Ppp1r3b*, and *Insig2*) in 52 gene products (Table S3), six genes (*Cyp7a1*, *Cyp7b1*, *Fasn*, *Sqle*, *Sc4mol*, and *Hmgcs1*) in 41 gene products (Table S4), and 4 genes (*Dhcr24*, *Akr1d1*, *Ldlr*, and *Cpt1a*) in 67 gene products (Table S5). The way in which each gene was regulated by the polyphenols (activation or suppression) was in accordance with the behavior of CAR activators [38]–[41]. Some of these genes were located at the critical steps of sugar or lipid metabolism, as described in the previous sections. We then examined the effect of the polyphenols on fatty liver progression in CAR-deficient mice. While CAR-deficient mice fed the ethanol diet developed AFL, as in the case of wild-type mice (Fig. 4A), the addition of the polyphenols to the diets had no effect on the fat accumulation, i.e., no significant difference between the ethanol group and the ethanol plus polyphenol groups (Fig. 4B). A liver transcriptome analysis of CAR-deficient mice revealed no segregation of the gene clusters between the ethanol group and the ethanol plus polyphenol groups (Fig. S2), in accordance with case of the phenotypes in the fat accumulation of the liver. In addition, most of the genes extracted by the gene ontology analysis were regulated in the way different from that of wild type mice; no biphasic change nor up-regulation in the presence of ethanol plus polyphenols diet compared to the control diet (Table S3 to S5).These results clearly indicate that the ameliorative effect of the polyphenols on the AFL is mediated by CAR.

## Discussion

In this study, we have analyzed changes in the liver transcriptome that correlates with the alleviation of AFL by the administration of the polyphenols occurring in alcoholic beverages. The resulting probe sets had 323, 287, 514, and 742 members, groups that were large enough to allow detection of gene enrichment in certain GO-term categories [30]. Although we processed the data with no preconceived hypothesis, the extracted genes showed some coordinated functions of metabolism that were in accordance with the previous studies on AFL. The predicted regulation of each metabolic pathway is discussed in the following sections.

Chronically administered ethanol causes production of unusually large amounts of acetic acid and NADH. This stream affects the TCA cycle and the other related pathways in two ways: by loading acetyl-CoA as a substrate and by providing excess NADH that inhibits NADH-producing steps in the pathways [11], [25], [26]. We found 10 gene products in these metabolic pathways showing coordinated regulation in response to the administration of ethanol and/or the polyphenols. In the presence of ethanol alone, five genes—*Ppp1r3b*, *Onecut1*, *Gck*, *Pgd*, and *Pdk*—were up-regulated. PPP1R3B inhibits glycogen synthesis by interacting with protein phosphatase 1 [42]. ONECUT1 (HNF6) stimulates transcription of glucose transporter 2 (*Glut2*) and *Gck* [43]. GCK and PGD are located at the critical steps of glycolysis and the pentose phosphate pathway, respectively [42], [44]. PDK4 phosphorylates pyruvate dehydrogenase (PD) complex, resulting in the inhibition of acetyl-CoA synthesis from pyruvate [45]. Augmentation of these gene products may lead to the inhibition of glycogen synthesis, the activation of glycolysis, activation of the pentose phosphate shunt, and inhibition of pyruvate incorporation to the TCA cycle, respectively (Fig. 3A). Under this condition, the acetyl-CoA derived from ethanol may be predominantly utilized for fatty acid synthesis with the supply of NADPH from the pentose phosphate pathway. Addition of the polyphenols to the diets suppressed the expression of all if these genes and induced the transcription of five other genes (*Slc37a4*, *Aco1*, *Idh2*, *FH1*, and *Mdh2*) that may facilitate the activation of gluconeogenesis and the facilitation of the TCA cycle (Fig. 3A). In summary, the polyphenols may suppress *de novo* lipogenesis from carbohydrates by activating gluconeogenesis, glycogen synthesis, and the TCA cycle and by inhibiting the pentose phosphate pathway under the condition of chronic ethanol feeding (Table 5).

**Table 5:** Summary of the predicted polyphenol's effect on metabolism.

Metabolic pathways	EtOH vs control	EtOH+EA or RSV vs EtOH	EtOH+EA or RSV vs control
*TCA cycle*	down	up	up
*Steoid and Bile acid synthesis* *	down	up	up
*β-oxidation* *	down	up	up
*α-oxidation*	down	up	up
*Tetrahydrofolate metabolism*	down	up	up
*Core clock (day)*	up	down	down
*Glycogen synthesis* *	down	up	
*Long chain unsaturated fatty acid synthesis*	down	up	
*Glycolysis*	up	down	
*Pentose phosphate pathway*	up	down	
*Gluconegenesis*			up
*NAD synthesis*			up
*Methionine metabolism*			up
*Urea cycle*			up
*Core clock (night)*			up
*Clock output regulators*			up

* : metabolic pathways that include CAR-regulated gene products.

Many researchers have reported that chronic ethanol consumption induces augmentation of fatty acid synthesis [25]–[27]. Contrary to this accepted idea, our transcriptomic analysis revealed that four enzyme genes involved in fatty acid synthesis were down-regulated by ethanol administration. These are *Acaca* and *Fasn*, located at the critical steps in *de novo* fatty acid synthesis (Fig. 3D), and *Elovl6* and *Fads2*, required for long-chain fatty acid elongation and desaturation, respectively (Fig. 3E) [46], [47]. Interestingly, addition of the polyphenols to the diets reversed the expression of all these genes and also induced *Oxsm*, whose product catalyzes incorporation of acetyl-CoA to the elongating fatty acid. Yin *et al.* [29] conducted liver transcriptomic analysis in mice fed ethanol for four weeks at low (3.6% of total calorie) and high (36% of total calorie) doses and found up-regulation of *Acaca* and *Fads2* at both doses. This finding partly contradicts our result, but the coordinated regulation of these 2 genes with the other genes (Fig. 3E and F) strongly suggests that the addition of the polyphenols to the diets may enhance the synthesis of long-chain unsaturated fatty acids. Song *et al.* [48] have reported that co-administration of long-chain polyunsaturated fatty acids (PUFAs) with ethanol decreased the hepatic triglyceride levels in rats compared with those fed an ethanol diet alone. They attributed this protective effect to the antioxidative activity of the long-chain PUFA. It is possible that the polyphenol's protective effect on fatty liver is partially mediated by the *de novo* PUFA synthesis.

As a component of the apolipoprotein complex, cholesterol plays a critical role in lipid transfer between the liver and peripheral tissues, and its amount is regulated by the balance among the food-derived intake, the *de novo* synthesis, and the excretion as bile acids [49]. We identified four genes (*Hmgcs1*, *Sqle*, *Tm7fs2*, and *Sc4mol*) for cholesterol synthesis and three genes (*Cyp7a1*, *Cyp7b1*, and *Hsd3b5*) for bile acid synthesis, all of which were down-regulated in the presence of ethanol alone (Fig. 3B). This overall suppression of cholesterol synthesis and its metabolism to bile acids seems to be a result of adaptive regulation by the excess supply of acetyl-CoA derived from ethanol [50]. However, addition of the polyphenols to the diets totally reversed this regulation, *i.e.*, the acceleration of this metabolic pathway. Additionally, two enzyme genes (*Dhcr24* and *Akr1d1*) and a receptor gene for cholesterol recovery from peripheral tissue (*Ldlr*) were found to be up-regulated in the presence of ethanol and the polyphenols. It can thus be concluded that the polyphenols promote the consumption of acetyl-CoA by *de novo* cholesterol synthesis, its recovery from peripheral tissue and its excretion in the form of bile acids [51].

Because there is no difference in fat composition between the ethanol diet and the control diet used in this study, the accumulation of triacylglycerol in the liver of the ethanol-administered mice can be attributable to overall imbalance between fatty acid synthesis and its catabolism. Our serum analysis revealed lower levels of triacylglycerol in mice administered ethanol or ethanol plus the polyphenols than those administered the control diet (Table S2). This suggests that the imbalance may occur within the liver, not between the liver and the peripheral tissues [12], [26], [52]. Under this condition, this may be an efficient way to accelerate fatty acid oxidation for reducing fat accumulation in the liver. Our finding that three enzyme genes for α-oxidation (*Slc27a2*, *Phyh*, and *Amacr*) and six enzyme genes for β-oxidation (*Acsm5*, *Cpt1a*, *Slc27a2*, *Acox1*, *Hsd17b4*, and *Acaa1a*) were up-regulated in the presence of the polyphenols (Fig. 3C) could be a rationale for the polyphenol's preventive effect on the AFL. In addition, it is possible that this regulation is a part of the metabolic response to xenobiotics, because xenobiotics, including polyphenols, can be oxidized in this peroxisomal pathway [36].

One of ethanol's adverse effects on the energy metabolism is the overproduction of NADH, which also means the depletion of its oxidized form, NAD. It is interesting to note that four enzyme genes (*Tdo2*, *Ido2*, *Afmid*, and *Kmo*) involved in the *de novo* synthesis of NAD and two enzyme genes (*Nampt* and *Nrk1*) involved in the nicotine amide salvage pathway were up-regulated only in the presence of ethanol plus the polyphenols (Fig. 3F). In addition, Kynu, whose product is located at the second-to-last step of the *de novo* synthesis pathway, showed up-regulation by feeding with ethanol alone and suppression by ethanol plus the polyphenols (Fig. 3F). These transcriptional regulations suggest that administration of the polyphenols may decline the *de novo* NAD synthesis and activate its salvage from the nicotine amide derivative's pool.

Metabolism of ethanol produces ROS, which have been recognized as some of the causative agents of liver steatosis. One of the endogenous protective mechanisms against ROS is the production of glutathione (GSH) as a reducing agent. The GSH metabolism is related to one-carbon metabolism through the precursor of this compound, homocysteine, which facilitates the production of methionine by receiving a methyl moiety from 5-methyl tetrahydrofolate (Fig. 3G). This pathway produces S-adenosyl methionine (SAM), which has also been shown to exert antioxidative effects in alcohol-fed rodents [53]. It is also important to mention that SAM provides a methyl moiety for the modification of various biomolecules, including deoxyribonucleic acid [54]. In our analysis, two enzyme genes (*ALdh1l1* and *Mthfd1*), whose products catalyze interconversion of tetrahydrofolate derivatives and three enzyme genes (*Mat2b*, *Bhmt*, and *Gnmt*) for methionine metabolism were found to be up-regulated in the presence of ethanol plus the polyphenols. In addition, *Dhfr* showed biphasic changes of the expression, *i.e.*, down-regulation by ethanol alone and up-regulation by ethanol plus the polyphenols (Fig. 3G). These regulations may facilitate utilization of folate for SAM and GSH production.

There is emerging evidence that nutritive status affects intrinsic oscillation of the clock genes both in the central nervous system (the suprachiasmatic nucleus, the SCN) and in the peripheral tissues [55]. The core clock consists of two groups of genes: the one expressed during daytime (*Bmal1* and *Clock*) and the other expressed at nighttime (*Per1, 2, 3*, *Cry1* and *2*). Interestingly, six of these components (*Arntl*, *Clock*, *Per1, 2, 3*, and *Cry2*) were found to be regulated by the polyphenol treatment (Fig. 3H). The polyphenols' effect seemed to be gene function selective; i.e., daytime genes were down-regulated, whereas nighttime genes were up-regulated (Fig. 3H). Whether or not the regulation of this type affects the phase and the amplitude of their oscillation should be examined as a future issue. However, it is notable that one of the clock genes, *Arntl* (*Bmal1*), showed biphasic regulation, *i.e.*, up-regulation by ethanol and down-regulation by ethanol plus the polyphenols. There are several reports about the impacts of ethanol administration on the central clock located in the SCN [56]. It is thus possible that the polyphenols antagonize the perturbation of the liver clock genes by ethanol administration by acting directly on the liver or *via* the SCN.

Ethanol is being recognized as a food factor that induces the epigenetic modification of genes because its administration alters the amounts of the key epigenetic regulatory factors, such as acetyl-CoA (excess production), NAD (depletion), and SAM (depletion) [12], [54]. These effects have been thought to be mediated mainly by ethanol metabolism, but our transcriptome analysis revealed that the significant changes also occurred in the expression of the enzyme genes involved in the production of these factors (Table 5). These transcriptional regulations may be accounted for by the adaptive regulation of the enzyme genes in response to the unusual supply of acetyl-CoA and NAD or to the need for endogenous antioxidative agents such as SAM and glutathione. Most noteworthy is the fact that the co-administration of the polyphenols markedly antagonized these regulations (Table 5). The precise contribution of these regulations to the amelioration of AFL needs to be elucidated in the context of metabolomics. However, this integrative effect of the polyphenols strongly suggests the presence of their molecular target, which is closely associated with transcriptional machinery. From this aspect, we have examined the dependence of the polyphenol's ameliorative effect on the nuclear receptor, CAR, and found that the deletion of the CAR gene extinguished the polyphenol's effect both phenotypically and transcriptomically (Fig. 4 and Fig. S2). It has been shown that the transcriptional regulation by nuclear receptors includes multiple steps of epigenetic modification at the gene loci under the control [19], [57], [58]. Accordingly, it is quite possible that CAR mediates the polyphenol's antagonistic effect against the epigenetic perturbation by ethanol administration at the transcriptional level. In conclusion, our study provides the molecular basis for the prevention of AFL by polyphenols, which are commonly consumed food factors.

## Supporting Information

Figure S1 **Induction of alcoholic fatty liver.** A, Feeding schedule of mice. Four mice for each experimental group were fed an MF diet for 1 week and then Lieber's diet with or without the polyphenols (Table S1) for 5 weeks under the conditions indicated below. B, Time course of average body weight. Groups fed an alcohol-containing diet showed less body weight than those fed the control diet. C, Relative liver weight of mice in each group. No significant differences were observed between the groups.
(PDF)

Figure S2 **Cluster analysis of the liver gene expression profiles of CAR-deficient mice fed under the conditions described in Fig. S1.** The data analysis procedure and the abbreviations are the same as in Fig. 2.
(PDF)

Table S1 **Formula of each diet (g).**
(PDF)

Table S2 **Analyses of serum markers.**
(PDF)

Table S3 **The list of 52 genes assigned to the GO-terms for 323 probe set.**
(PDF)

Table S4 **The list of 41 genes assigned to the GO-terms for 287 probe set.**
(PDF)

Table S5 **The list of 67 genes assigned to the GO-terms for 514 probe set.**
(PDF)

Table S6 **The list of 253 genes assigned to the GO-terms for 742 probe set.**
(PDF)
