# Supplementary figures and images for: Nuclear Receptor-Mediated Alleviation of Alcoholic Fatty Liver by Polyphenols Contained in Alcoholic Beverages
Source: PLoS One. 2014 Feb 3;9(2):e87142. doi: 10.1371/journal.pone.0087142 (PMC3911942; doi:10.1371/journal.pone.0087142)

A

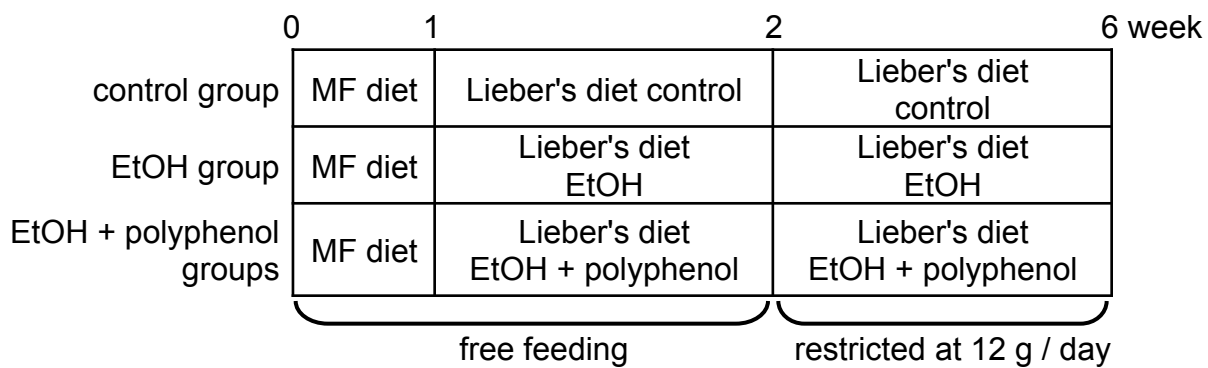

B

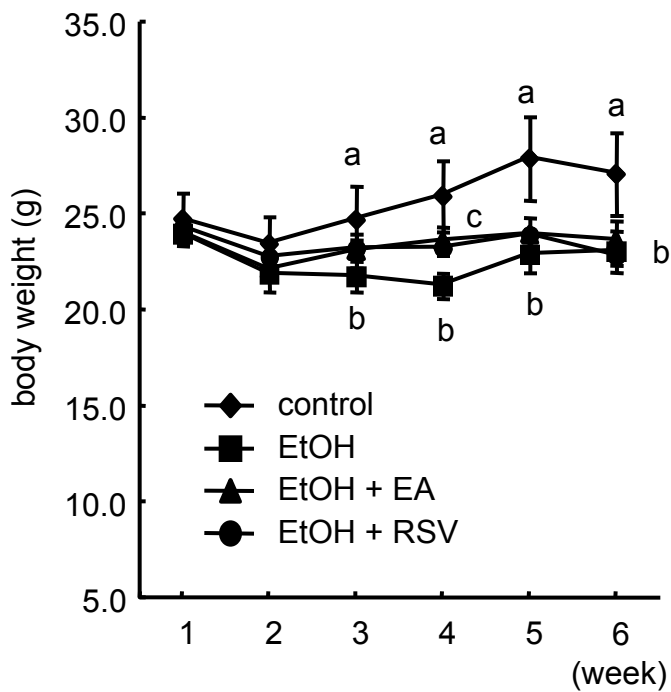

C

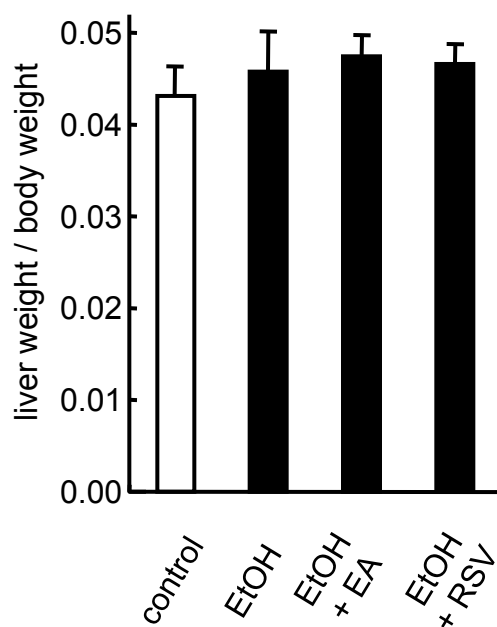

Supplement: Figure S1 — Induction of alcoholic fatty liver. A, Feeding schedule of mice. Four mice for each experimental group were fed an MF diet for 1 week and then Lieber's diet with or without the polyphenols (Table S1) for 5 weeks under the conditions indicated below. B, Time course of average body weight. Groups fed an alcohol-containing diet showed less body weight than those fed the control diet. C, Relative liver weight of mice in each group. No significant differences were observed between the groups. (PDF) [file pone.0087142.s001.pdf]

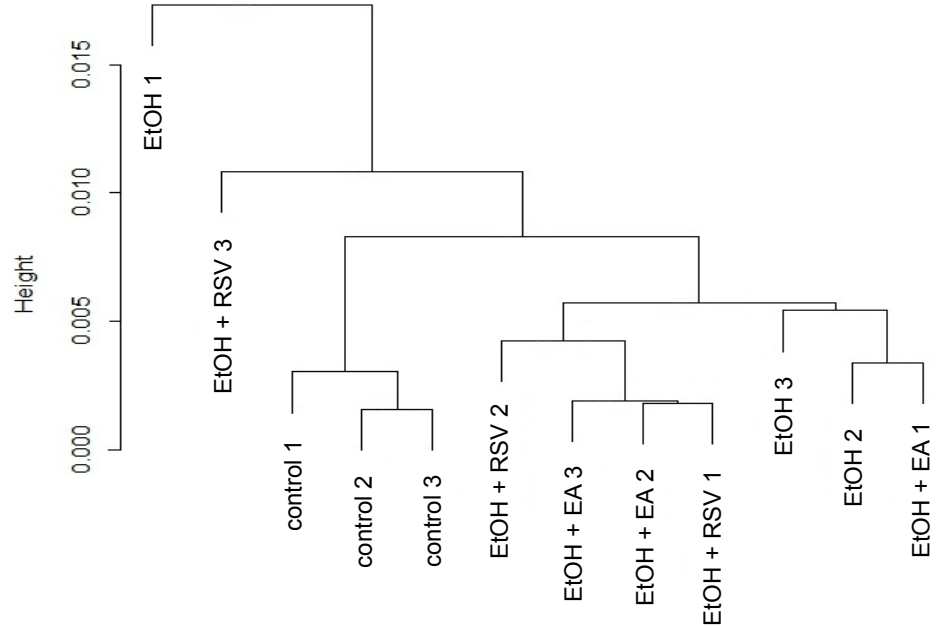

Supplement: Figure S2 — Cluster analysis of the liver gene expression profiles of CAR-deficient mice fed under the conditions described in Fig. S1. The data analysis procedure and the abbreviations are the same as in Fig. 2. (PDF) [file pone.0087142.s002.pdf]
