# Supplementary material for: Nuclear Receptor-Mediated Alleviation of Alcoholic Fatty Liver by Polyphenols Contained in Alcoholic Beverages
Source: PLoS One. 2014 Feb 3;9(2):e87142. doi: 10.1371/journal.pone.0087142 (PMC3911942; doi:10.1371/journal.pone.0087142)
Supplement: Table S1 — Formula of each diet (g). (PDF) [file pone.0087142.s003.pdf]

Table S1. Formula of each diet (g)

	control	EtOH	EtOH + polyphenol
casein sodium salt	41.4	41.4	41.4
l - cystine	0.5	0.5	0.5
dl - methionine	0.3	0.3	0.3
corn oil	8.5	8.5	8.5
olive oil	28.4	28.4	28.4
safflower oil	2.7	2.7	2.7
vitamin mix	2.5	2.5	2.5
mineral mix	8.75	8.75	8.75
mixture of equal parts of maltose and dextrin	115.2	25.6	25.6
cellulose	10.0	10.0	10.0
choline bitartrate	0.53	0.53	0.53
xanthane gum	3.0	3.0	3.0
total	221.78	182.18	182.18
ethanol	0 mL	50 mL	50 mL
water	1000 mL	950 mL	950 mL
ellagic acid or resveratrol	0	0	73 mg
