# Supplementary material for: Nuclear Receptor-Mediated Alleviation of Alcoholic Fatty Liver by Polyphenols Contained in Alcoholic Beverages
Source: PLoS One. 2014 Feb 3;9(2):e87142. doi: 10.1371/journal.pone.0087142 (PMC3911942; doi:10.1371/journal.pone.0087142)
Supplement: Table S2 — Analyses of serum markers. (PDF) [file pone.0087142.s004.pdf]

Table S2. Analyses of serum markers

| | genotype | control | EtOH | EtOH + EA | EtOH + RSV |
|---|---|---|---|---|---|
| total cholesterol (mg/dL) | WT | 174±10.0 | 187±11.2 | 191±15.3 | 193±10.1 |
| | CAR KO | 150±27.0 | 194±11.0 | 196±17.5 | 182±24.2 |
| triacylglycerol (mg/dL) | WT | 91.8±14.1 **a** | 60.5±9.88 **b** | 54.8±9.50 **b** | 42.5±11.8 **b** |
| | CAR KO | 37.3±8.08 | 52.7±23.9 | 57.3±4.73 | 41.7±13.2 |
| non-esterified free fatty acid (μEq/L) | WT | 2100±832 | 1360±374 | 1330±215 | 1270±126 |
| | CAR KO | 1530±356 | 1390±207 | 1530±359 | 1160±163 |
| total bilirubin (mg/dL) | WT | 0.0925±0.0150 | 0.157±0.0768 | 0.0875±0.0299 | 0.107±0.0189 |
| | CAR KO | 0.130±0.0300 | 0.206±0.0642 | 0.143±0.00577 | 0.170±0.0436 |
| glucose (mg/dL) | WT | 144±32.9 | 128±32.0 | 154±43.5 | 139±20.1 |
| | CAR KO | 107±55.5 | 61.7±26.4 | 118±25.1 | 102±55.2 |
| total ketone bod (μmol/L) | WT | 433±166 | 913.±281 | 1030±384 | 1050±370 |
| | CAR KO | 520±68.8 | 1430±711 | 1410±322 | 1430±877 |
| HDL-cholesterol (mg/dL) | WT | 102±2.06 **a** | 117±11.4 **ab** | 126±7.66 **b** | 118±5.74 **b** |
| | CAR KO | 88.3±12.5 | 117±18.9 | 116±11.2 | 101±7.00 |
| LDL-cholesterol (mg/dL) | WT | 8.50±1.91 | 8.00±1.63 | 7.75±2.87 | 7.50±0.58 |
| | CAR KO | 10.0±2.00 | 7.67±1.53 | 9.67±2.00 | 12.3±4.51 |

a and b: Tukey's multiple comparison between control, EtOH, EtOH + EA and EtOH + RSV groups each of which contains 4 (WT) or 3 (CAR KO) mice
