# Supplementary material for: Nuclear Receptor-Mediated Alleviation of Alcoholic Fatty Liver by Polyphenols Contained in Alcoholic Beverages
Source: PLoS One. 2014 Feb 3;9(2):e87142. doi: 10.1371/journal.pone.0087142 (PMC3911942; doi:10.1371/journal.pone.0087142)
Supplement: Table S3 — The list of 52 genes assigned to the GO-terms for 323 probe set. (PDF) [file pone.0087142.s005.pdf]

Table S3. The list of 52 genes assigned to the GO-terms for 323 probe set

Gene name	Gene symbol	probe ID	Mapped panel in Fig.3	Reported to be regulated by CAR	Similary regulated in CAR KO mice
**GO:0006986~response to unfolded protein**					
DnaJ (Hsp40) homolog, subfamily C, member 3	Dnajc3	1419162_S_AT<br>1433887_AT<br>1419163_S_AT<br>1449372_AT			
UBX domain protein 4	Ubxn4	1426486_AT<br>1426485_AT			
mesencephalic astrocyte-derived neurotrophic factor	Manf	1428112_AT			
predicted gene 5511; heat shock protein 90, alpha (cytosolic), class A member 1	Hsp90aa1	1426645_AT<br>1437497_A_AT<br>1438902_A_AT			
stress-associated endoplasmic reticulum protein 1	Serp1*	1415827_A_AT			
synovial apoptosis inhibitor 1, synoviolin	Syvn1	1443609_S_AT<br>1428070_AT			
**GO:0043161~proteasomal ubiquitin-dependent protein catabolic process**					
STIP1 homology and U-Box containing protein 1	Stub1*	1416580_A_AT			
predicted gene 3375; proteasome (prosome, macropain) subunit, beta type 5	Psmb5	1415676_A_AT			
protease (prosome, macropain) 26S subunit, ATPase 5	Psmc5*	1415740_AT			
protein phosphatase 2, regulatory subunit B (B56), gamma isoform	Ppp2r5c	1425542_A_AT			
**GO:0031016~pancreas development**					
X-box binding protein 1	Xbp1	1420011_S_AT<br>1420886_A_AT<br>1437223_S_AT			
immunoglobulin-like domain containing receptor 2	Ildr2	1436221_AT<br>1436293_X_AT			
one cut domain, family member 1	Onecut1*	1450252_AT	A		
one cut domain, family member 2	Onecut2*	1460044_AT			
**GO:0051246~regulation of protein metabolic process**					
Jun oncogene	Jun*	1448694_AT<br>1417409_AT			
STIP1 homology and U-Box containing protein 1	Stub1*	1416580_A_AT			
aryl hydrocarbon receptor nuclear translocator-like	Arntl (Bmal1)*	1425099_A_AT	H		
cell division cycle 34 homolog (S. cerevisiae); similar to Cell division cycle 34 homolog (S. cerevisiae)	Cdc34	1434879_AT			
cystathionine beta-synthase	Cbs	1425623_A_AT<br>1423844_S_AT	I		

cytoplasmic polyadenylation element binding protein 2	Cpeb2	1443017_AT<br>1458518_AT<br>1434272_AT			+
imprinted and ancient	Impact *	1415911_AT			
signal recognition particle 9	Srp9*	1417530_A_AT			
stress-associated endoplasmic reticulum protein 1	Serp1*	1415827_A_AT			
suppressor of cytokine signaling 3	Socs3	1456212_X_AT<br>1455899_X_AT			
ubiquitin-conjugating enzyme E2A, RAD6 homolog (S. cerevisiae)	Ube2a	1448772_AT			
zinc finger protein 36, C3H type-like 1	Zfp36l1	1422528_A_AT			
**GO:0051085~chaperone mediated protein folding requiring cofactor**					
DnaJ (Hsp40) homolog, subfamily B, member 1	Dnajb1	1416756_AT			
heat shock 105kDa/110kDa protein 1	Hsph1	1423566_A_AT<br>1425993_A_AT			
similar to heat shock protein 8; heat shock protein 8	Hspa8	1420622_A_AT<br>1420623_X_AT<br>1455789_X_AT			
**GO:0006006~glucose metabolic process**					
glucokinase	Gck *	1419146_A_AT<br>1425303_AT	A		
myelocytomatosis oncogene	Myc *	1424942_A_AT			
one cut domain, family member 1	Onecut 1*	1450252_AT	A		
phosphogluconate dehydrogenase	Pgd *	1437380_X_AT<br>1436771_X_AT<br>1438627_X_AT	A	+	
protein phosphatase 1, regulatory (inhibitor) subunit 3B	Ppp1r3 b	1436590_AT	A	+	
pyruvate dehydrogenase kinase, isoenzyme 4	Pdk4	1417273_AT	A		
stress-associated endoplasmic reticulum protein 1	Serp1*	1415827_A_AT			
**GO:0043603~cellular amide metabolic process**					
argininosuccinate synthetase 1	Ass1	1459937_AT			
glucokinase	Gck *	1419146_A_AT<br>1425303_AT	A		
kynureninase (L-kynurenine hydrolase)	Kynu	1430570_AT	F		
phosphogluconate dehydrogenase	Pgd *	1436771_X_AT<br>1437380_X_AT<br>1438627_X_AT	A	+	
**GO:0031324~negative regulation of cellular metabolic process**					
Jun oncogene	Jun *	1448694_AT<br>1417409_AT			
Kruppel-like factor 11	Klf11	1437241_AT			
forkhead box P1	Foxp1	1455242_AT<br>1421141_A_AT			
glucokinase	Gck *	1419146_A_AT<br>1425303_AT	A		
imprinted and ancient	Impact *	1415911_AT			
inhibitor of DNA binding 2	Id2	1422537_A_AT			
insulin induced gene 2	Insig2	1417981_AT	B	+	

		1417980_A_AT			
interferon regulatory factor 2 binding protein 2	Irf2bp2	1433633_AT 1433634_AT 1433632_AT			+
myelocytomatosis oncogene	Myc *	1424942_A_AT			
nuclear receptor interacting protein 1	Nrip1	1434384_AT			
protease (prosome, macropain) 26S subunit, ATPase 5	Psmc5*	1415740_AT			
signal recognition particle 9	Srp9*	1417530_A_AT			
similar to cytoplasmic dynein light chain 1; predicted gene 11582; dynein light chain LC8-type 1; predicted gene 6788	Dynll1	1417339_A_AT			
transducin-like enhancer of split 4, homolog of Drosophila E(spl)	Tle4	1450853_AT			
zinc finger and BTB domain containing 16	Zbtb16	1442025_A_AT 1427638_AT 1419874_X_AT 1442026_AT 1439163_AT			+
**GO:0006886~intracellular protein transport**					
RAB1, member RAS oncogene family	Rab1	1416082_AT			
Sec24 related gene family, member D (S. cerevisiae)	Sec24d	1426972_AT			
Sec61 alpha 1 subunit (S. cerevisiae)	Sec61a 1	1416190_A_AT 1416189_A_AT 1448242_AT 1434986_A_AT			
aryl hydrocarbon receptor nuclear translocator-like	Arntl (Bmal1) *	1425099_A_AT	H		
coatomer protein complex, subunit gamma	Copg	1416017_AT 1415670_AT			
histocompatibility 47	H47	1448704_S_AT 1435735_X_AT 1452095_A_AT			
predicted gene, EG546165; predicted gene 2423; hypothetical protein LOC674211; tyrosine 3-monooxygenase/tryptophan 5-monooxygenase activation protein, theta polypeptide	Ywhaq	1432842_S_AT			
signal recognition particle 9	Srp9*	1417530_A_AT			
transmembrane emp24-like trafficking protein 10 (yeast); predicted gene 4024	Tmed10	1424707_AT			
tyrosine 3-monooxygenase/tryptophan 5-monooxygenase activation protein, beta polypeptide	Ywhab	1436783_X_AT			
**GO:0030512~negative regulation of transforming growth factor beta receptor signaling pathway**					
heat shock protein 5	Hspa5	1447824_X_AT 1416064_A_AT			

		1427464_S_AT			
one cut domain, family member 1	Onecut 1*	1450252_AT	A		
one cut domain, family member 2	Onecut 2*	1460044_AT			

* : Genes appearing more than two times in the functional groupings.
