# Supplementary material for: Nuclear Receptor-Mediated Alleviation of Alcoholic Fatty Liver by Polyphenols Contained in Alcoholic Beverages
Source: PLoS One. 2014 Feb 3;9(2):e87142. doi: 10.1371/journal.pone.0087142 (PMC3911942; doi:10.1371/journal.pone.0087142)
Supplement: Table S4 — The list of 41 genes assigned to the GO-terms for 287 probe set. (PDF) [file pone.0087142.s006.pdf]

Table S4. The list of 41 genes assigned to the GO-terms for 287 probe set

Gene name	Gene symbol	probe ID	Mapped panel in Fig.3	Reported to be regulated by CAR	Similary regulated in CAR KO mice
**GO:0055114~oxidation reduction**					
2-4-dienoyl-Coenzyme A reductase 2, peroxisomal	Decr2	1423495_AT			
WW domain-containing oxidoreductase	Wwox	1446412_AT 1431960_AT			
butyrobetaine (gamma), 2-oxoglutarate dioxygenase 1 (gamma-butyrobetaine hydroxylase)	Bbox1	1459030_AT			
cDNA sequence BC089597	BC089597 (retinol dehydrogenase)	1451681_AT			+
cytochrome P450, family 2, subfamily d, polypeptide 9	Cyp2d9	1419349_A_AT			+
cytochrome P450, family 7, subfamily a, polypeptide 1	Cyp7a1*	1422100_AT 1438743_AT	B	+	+
cytochrome P450, family 7, subfamily b, polypeptide 1	Cyp7b1*	1421074_AT 1421075_S_AT	B	+	
dihydrofolate reductase	Dhfr	1419172_AT	G		
fatty acid desaturase 2	Fads2*	1419031_AT 1443838_X_AT	E		
fatty acid synthase	Fasn *	1423828_AT	D	+	
gulonolactone (L-) oxidase	Gulo	1451297_AT			
hydroxy-delta-5-steroid dehydrogenase, 3 beta- and steroid delta-isomerase 5	Hsd3b5*	1420531_AT	B		
retinol dehydrogenase 11	Rdh11	1449209_A_AT			
retinol dehydrogenase 14 (all-trans and 9-cis)	Rdh14	1417438_AT			
short chain dehydrogenase/reductase family 42E, member 1	Sdr42e1*	1427082_AT			
squalene epoxidase	Sqle	1415993_AT	B	+	
sterol-C4-methyl oxidase-like	Sc4mol *	1423078_A_AT	B	+	
transmembrane 7 superfamily member 2	Tm7sf2 *	1460684_AT	B		
**GO:0006694~steroid biosynthetic process**					
alpha-methylacyl-CoA racemase	Amacr	1417208_AT	C		
cytochrome P450, family 7, subfamily b, polypeptide 1	Cyp7b1*	1421074_AT 1421075_S_AT	B		
hydroxy-delta-5-steroid dehydrogenase, 3 beta- and steroid delta-isomerase 5	Hsd3b5*	1420531_AT	B		
short chain	Sdr42e1*	1427082_AT			

dehydrogenase/reductase family 42E, member 1					
similar to Hmgcs1 protein; 3-hydroxy-3-methylglutaryl-Coenzyme A synthase 1	Hmgcs1*	1433445_X_AT 1433443_A_AT	B	+	
sterol-C4-methyl oxidase-like	Sc4mol *	1423078_A_AT	B	+	
transmembrane 7 superfamily member 2	Tm7sf2*	1460684_AT	B		
**GO:0008203~cholesterol metabolic process**					
cytochrome P450, family 7, subfamily a, polypeptide 1	Cyp7a1*	1438743_AT 1422100_AT	B	+	+
cytochrome P450, family 7, subfamily b, polypeptide 1	Cyp7b1*	1421074_AT 1421075_S_AT	B	+	
serum amyloid A 1	Saa1	1450788_AT 1419075_S_AT		+	+
similar to Hmgcs1 protein; 3-hydroxy-3-methylglutaryl-Coenzyme A synthase 1	Hmgcs1*	1433443_A_AT 1433445_X_AT	B	+	
transmembrane 7 superfamily member 2	Tm7sf2 *	1460684_AT	B		
**GO:0006633~fatty acid biosynthetic process**					
fatty acid desaturase 2	Fads2*	1419031_AT 1443838_X_AT	E		
fatty acid synthase	Fasn *	1423828_AT	D	+	
predicted gene 11295; ELOVL family member 6, elongation of long chain fatty acids (yeast)	Elovl6	1445062_AT 1417404_AT 1417403_AT	D		
predicted gene 5182; acetyl-Coenzyme A carboxylase alpha	Acaca	1434185_AT	D		
sterol-C4-methyl oxidase-like	Sc4mol *	1423078_A_AT	B	+	
**GO:0006869~lipid transport**					
apolipoprotein L 7a	Apol7a	1453080_AT			
oxysterol binding protein-like 3	Osbpl3 (Orp3)	1438724_AT 1428484_AT			
phosphatidylinositol transfer protein, cytoplasmic 1	Pitpnc1 (RdgB)	1441574_AT			
phospholipid transfer protein	Pltp	1456424_S_AT			
solute carrier family 27 (fatty acid transporter), member 2	Slc27a2 (Fatp2)	1416316_AT	C		
spinster homolog 2 (Drosophila)	Spns2*	1451601_A_AT			
**GO:0055085~transmembrane transport**					
ATPase, H+ transporting, lysosomal V0 subunit D2	Atp6v0d2	1434798_AT			
aquaporin 4	Aqp4	1434449_AT			
predicted gene 7180; translocase of inner mitochondrial membrane	Timm17a	1426256_AT			

17a					
predicted gene 9797; translocase of inner mitochondrial membrane 8 homolog a1 (yeast); similar to small zinc finger-like protein	Timm8a1	1416345_AT			
scavenger receptor class A, member 5 (putative)	Scara5 (Ferritin transporter)	1451204_AT			
similar to X-linked PEST-containing transporter; solute carrier family 16 (monocarboxylic acid transporters), member 2	Slc16a2 (Mct8, thyroid hormone transporter)	1418445_AT			
solute carrier family 12, member 7	Slc12a7 (Kcc1, K / Cl transporter)	1418257_AT			
solute carrier family 16 (monocarboxylic acid transporters), member 10	Slc16a10 (Mct10, thyroid hormone transporter)	1457851_AT			
solute carrier family 22 (organic anion transporter), member 7	Slc22a7 (Oat2, uric acid transporter)	1451460_A_AT			+
solute carrier family 22, member 23	Slc22a23 (Boct2)	1453004_AT			
solute carrier family 23 (nucleobase transporters), member 1	Slc23a1 ascorbic acid transporter)	1421912_AT			
solute carrier family 26 (sulfate transporter), member 1	Slc26a1 (sulfuriic acid transporter)	1451239_A_AT 1458327_X_AT			
spinster homolog 2 (Drosophila)	Spns2*	1451601_A_AT			

* : Genes appearing more than two times in the functional groupings.
