# Supplementary material for: Nuclear Receptor-Mediated Alleviation of Alcoholic Fatty Liver by Polyphenols Contained in Alcoholic Beverages
Source: PLoS One. 2014 Feb 3;9(2):e87142. doi: 10.1371/journal.pone.0087142 (PMC3911942; doi:10.1371/journal.pone.0087142)
Supplement: Table S5 — The list of 67 genes assigned to the GO-terms for 514 probe set. (PDF) [file pone.0087142.s007.pdf]

Table S5. The list of 67 genes assigned to the GO-terms for 514 probe set

Gene name	Gene symbol	probe ID	Mapped panel in Fig.3	Reported to be regulated by CAR	Similary regulated in CAR KO mice
**GO:0007623~circadian rhythm**					
D site albumin promoter binding protein	Dbp	1418174_AT<br>1438211_S_AT	H		+
period homolog 1 (Drosophila)	Per1	1449851_AT	H		
period homolog 2 (Drosophila)	Per2	1417602_AT	H		
period homolog 3 (Drosophila)	Per3	1458176_AT<br>1441445_AT<br>1442243_AT<br>1421087_AT	H		
similar to mKIAA0658 protein; cryptochrome 2 (photolyase-like)	Cry2	1426383_AT	H		
**GO:0008202~steroid metabolic process**					
24-dehydrocholesterol reductase	Dhcr24	1451895_A_AT	B	+	
StAR-related lipid transfer (START) domain containing 5	Stard5	1422822_AT<br>1450769_S_AT			
aldo-keto reductase family 1, member D1	Akr1d1	1455100_AT<br>1425771_AT	B	+	
ceroid-lipofuscinosis, neuronal 8	Cln8	1448455_AT			
low density lipoprotein receptor	Ldlr	1421821_AT		+	
nuclear receptor subfamily 3, group C, member 1	Nr3c1(GR)*	1460303_AT<br>1457635_S_AT			
oxysterol binding protein-like 1A	Osbpl1a	1460192_AT			
oxysterol binding protein-like 3	Osbpl3	1428484_AT<br>1438724_AT			
serine (or cysteine) peptidase inhibitor, clade A, member 6	Serpina6	1448506_AT			
solute carrier family 37 (glucose-6-phosphate transporter), member 4	Slc37a4	1417042_AT	A		
**GO:0043603~cellular amide metabolic process**					
carbamoyl-phosphate synthetase 1	Cps1*	1455540_AT	I		
dicarbonyl L-xylulose reductase	Dcxr	1419456_AT			
kynurenine 3-monooxygenase (kynurenine 3-hydroxylase)	Kmo*	1418998_AT	F		
nicotinamide phosphoribosyltransferase	Nampt	1455320_AT	F		
ornithine transcarbamylase	Otc*	1436615_A_AT	I		
**GO:0044271~nitrogen compound biosynthetic process**					
ATP synthase, H+	Atp5c1	1438809_AT			

transporting, mitochondrial F1 complex, gamma polypeptide 1					
GTP cyclohydrolase 1	Gch1*	1429692_S_AT			
adenosine kinase	Adk	1438292_X_AT			
antizyme inhibitor 1	Azin1	1450714_AT			+
betaine-homocystein e methyltransferase	Bhmt*	1450624_AT	G		
cDNA sequence BC016495(Nrk1)	BC0164 95 (Nrk1)*	1425646_AT	F		
carbamoyl-phosphate synthetase 1	Cps1*	1455540_AT	I		
dihydropyrimidine dehydrogenase	Dpyd	1427945_AT			
ethanolamine kinase 2; similar to putative ethanolamine kinase	Etnk2	1437040_AT			
kynurenine 3-monooxygenase (kynurenine 3-hydroxylase)	Kmo*	1418998_AT	F		
methionine adenosyltransferase II, beta	Mat2b*	1448196_AT	G		
methylenetetrahydrof olate dehydrogenase (NADP+ dependent), methenyltetrahydrofol ate cyclohydrolase, formyltetrahydrofolate synthase	Mthfd1*	1436704_X_AT 1415917_AT	G		
nicotinamide phosphoribosyltransf erase	Nampt*	1455320_AT	F		
ornithine transcarbamylase	Otc*	1436615_A_AT	I		
phosphoribosylaminoi midazole carboxylase, phosphoribosylamino ribosylaminoimidazol e, succinocarboxamide synthetase; predicted gene 8445	Paics	1436298_X_AT			
**GO:0042364~water-soluble vitamin biosynthetic process**					
cDNA sequence BC016495(Nrk1)	BC0164 95 (Nrk1)*	1425646_AT			
kynurenine 3-monooxygenase (kynurenine 3-hydroxylase)	Kmo*	1418998_AT	F		
nicotinamide phosphoribosyltransf erase	Nampt*	1455320_AT	F		
pyridoxal (pyridoxine, vitamin B6) kinase	Pdxk	1427929_A_AT 1427931_S_AT			
**GO:0009108~coenzyme biosynthetic process**					
GTP cyclohydrolase	Gch1*	1429692_S_AT			

1					
cDNA sequence BC016495(Nrk1)	BC0164 95 (Nrk1)*	1425646_AT	F		
dihydrolipoamide branched chain transacylase E2	Dbt	1449118_AT			
kynurenine 3-monooxygenase (kynurenine 3-hydroxylase)	Kmo*	1418998_AT	F		
methylenetetrahydrofolate dehydrogenase (NADP+ dependent), methenyltetrahydrofolate cyclohydrolase, formyltetrahydrofolate synthase	Mthfd1*	1415917_AT 1436704_X_AT	G		
nicotinamide phosphoribosyltransferase	Nampt*	1455320_AT	F		
**GO:0030203~glycosaminoglycan metabolic process**					
decorin	Dcn	1449368_AT			
glucosamine (N-acetyl)-6-sulfatase	Gns	1433488_X_AT			
hexosaminidase B	Hexb	1437874_S_AT			
hyaluronoglucosaminidase 2	Hyal2	1448679_AT			
inter-alpha (globulin) inhibitor H5	Itih5	1436755_AT			
**GO:0046653~tetrahydrofolate metabolic process**					
GTP cyclohydrolase 1	Gch1*	1429692_S_AT			
pipecolic acid oxidase	Pipox	1449374_AT			
similar to Aldehyde dehydrogenase 1 family, member L1; aldehyde dehydrogenase 1 family, member L1	Aldh1l1	1424400_A_AT	G		
**GO:0006631~fatty acid metabolic process**					
3-oxoacyl-ACP synthase, mitochondrial	Oxsm	1455395_AT	D		
NADH dehydrogenase (ubiquinone) 1, alpha/beta subcomplex, 1; predicted gene 4459	Ndufab1	1447919_X_AT			
acetyl-Coenzyme A acyltransferase 1A	Acaa1a	1424451_AT 1416947_S_AT 1456011_X_AT	C		
acyl-CoA synthetase medium-chain family member 5	Acsm5	1437662_AT	C		
acyl-Coenzyme A oxidase 1, palmitoyl	Acox1	1416409_AT 1416408_AT	C	+ opposite	
carnitine palmitoyltransferase 1a, liver	Cpt1a	1434866_X_AT 1438156_X_AT	C	+	
cytochrome P450, family 2, subfamily d,	Cyp2d2 2	1419039_AT 1419040_AT			

polypeptide 22					
fatty acid desaturase domain family, member 6	Fads6	1443904_AT			
hydroxysteroid (17-beta) dehydrogenase 4	Hsd17b4	1455777_X_AT	C		
peroxisome proliferator activated receptor alpha	Ppara	1457721_AT<br>1439675_AT<br>1449051_AT			
phytanoyl-CoA hydroxylase	Phyh	1460194_AT	C		
protein kinase, AMP-activated, gamma 2 non-catalytic subunit	Prkag2	1423831_AT			
**GO:0006099~tricarboxylic acid cycle**					
aconitase 1	Aco1	1456728_X_AT	A		
fumarate hydratase 1	Fh1	1424828_A_AT	A		
isocitrate dehydrogenase 2 (NADP+), mitochondrial	Idh2	1458383_AT	A		
malate dehydrogenase 2, NAD (mitochondrial)	Mdh2	1433984_A_AT	A		
**GO:0006446~regulation of translational initiation**					
ankyrin repeat and KH domain containing 1; eukaryotic translation initiation factor 4E binding protein 3	Ankhd1/Eif4ebp3	1453023_AT			
poly(A) binding protein interacting protein 2B	Paip2b	1451125_AT			
protein phosphatase 1, regulatory (inhibitor) subunit 15b	Ppp1r15b	1426798_A_AT			
similar to Gene model 672, (NCBI); predicted gene 672	Gm672	1436047_AT			
**GO:0006555~methionine metabolic process**					
betaine-homocysteine methyltransferase	Bhmt*	1450624_AT	G		
glycine N-methyltransferase	Gnmt*	1417422_AT	G		
methylenetetrahydrofolate dehydrogenase (NADP+ dependent), methenyltetrahydrofolate cyclohydrolase, formyltetrahydrofolate synthase	Mthfd1*	1436704_X_AT<br>1415917_AT	G		
**GO:0046500~S-adenosylmethionine metabolic process**					
glycine N-methyltransferase	Gnmt*	1417422_AT	G		
methionine adenosyltransferase II, beta	Mat2b*	1448196_AT	G		
thiopurine methyltransferase	Tpmt	1456273_X_AT<br>1438087_AT	G		
**GO:0006111~regulation of gluconeogenesis**					

glycine N-methyltransferase	Gnmt*	1417422_AT	G		
hepatic nuclear factor 4, alpha	Hnf4a	1427000_AT			
nuclear receptor subfamily 3, group C, member 1	Nr3c1(GR)*	1457635_S_AT<br>1460303_AT			
**GO:0019441~tryptophan catabolic process to kynurenine**					
arylformamidase	Afmid	1428885_AT<br>1452944_AT	F		
indoleamine 2,3-dioxygenase 2	Ido2	1425778_AT	F		
tryptophan 2,3-dioxygenase	Tdo2	1455770_AT	F		

* : Genes appearing more than two times in the functional groupings.
