# Supplementary material for: Nuclear Receptor-Mediated Alleviation of Alcoholic Fatty Liver by Polyphenols Contained in Alcoholic Beverages
Source: PLoS One. 2014 Feb 3;9(2):e87142. doi: 10.1371/journal.pone.0087142 (PMC3911942; doi:10.1371/journal.pone.0087142)
Supplement: Table S6 — The list of 253 genes assigned to the GO-terms for 742 probe set. (PDF) [file pone.0087142.s008.pdf]

Table S6. The list of 253 genes assigned to the GO-terms for 742 probe set

Gene name	Gene symbol	probe ID
**GO:0044087~regulation of cellular component biogenesis**		
KN motif and ankyrin repeat domains 2	Kank2	1425991_A_AT
actin related protein 2/3 complex, subunit 1B	Arpc1b	1416226_AT
agrin	Agrn*	1426670_AT
capping protein (actin filament) muscle Z-line, alpha 1; similar to capping protein (actin filament) muscle Z-line, alpha 1; predicted gene 3608; predicted gene 5920	Capza1	1439455_X_AT<br>1452038_AT
phosphatase and tensin homolog	Pten*	1455728_AT<br>1422553_AT
predicted gene 5492; actin related protein 2/3 complex, subunit 2	Arpc2	1437148_AT
predicted gene 8545; microtubule-associated protein, RP/EB family, member 1; similar to Microtubule-associated protein RP/EB family member 1 (APC-binding protein EB1) (End-binding protein 1) (EB1)	Mapre1	1450740_A_AT<br>1422764_AT
similar to adenomatosis polyposis coli; adenomatosis polyposis coli	Apc	1435543_AT
tropomodulin 3	Tmod3	1438556_A_AT
**GO:0043687~post-translational protein modification**		
B-cell translocation gene 1, anti-proliferative; similar to myocardial vascular inhibition factor	Btg1*	1426083_A_AT
Bmi1 polycomb ring finger oncogene	Bmi1*	1417493_AT
CREB binding protein	Crebbp*	1436983_AT
ERO1-like (S. cerevisiae)	Ero1l*	1449324_AT
ERO1-like beta (S. cerevisiae)	Ero1lb*	1434714_AT<br>1425705_A_AT
F-box protein 4	Fbxo4*	1427121_AT
G1 to S phase transition 1	Gspt1*	1452168_X_AT<br>1426736_AT
HECT, UBA and WWE domain containing 1	Huwe1*	1415703_AT
OTU domain containing 7B	Otud7b	1429139_AT
PCTAIRE-motif protein kinase 1	Pctk1	1438625_S_AT
PDLIM1 interacting kinase 1 like	Pdik1l	1437175_AT
PEST proteolytic signal containing nuclear protein	Pcnp*	1452735_AT
SMT3 suppressor of mif two 3 homolog 3 (yeast)	Sumo3	1422457_S_AT
TAO kinase 1	Taok1	1424657_AT
adrenergic receptor kinase, beta 1	Adrbk1	1451992_AT
cAMP responsive element binding protein 1	Creb1*	1452901_AT
coagulation factor II (thrombin) receptor	F2r	1437308_S_AT
dimethylarginine dimethylaminohydrolase 1	Ddah1	1455400_AT<br>1429299_AT<br>1454995_AT
dual specificity phosphatase 1	Dusp1	1448830_AT
dual specificity phosphatase 11 (RNA/RNP complex 1-interacting)	Dusp11	1452594_AT
eukaryotic translation termination factor 1	Etf1*	1451208_AT
histone deacetylase 2	Hdac2*	1445684_S_AT
interleukin-1 receptor-associated kinase 2	Irak2	1436507_AT
myeloid/lymphoid or mixed-lineage leukemia 5	Mll5*	1439108_AT
nuclear receptor-binding SET-domain protein 1	Nsd1*	1435088_AT
phosphatase and tensin homolog	Pten*	1422553_AT<br>1455728_AT
predicted gene 13416; WW domain containing E3 ubiquitin protein ligase 1	Wwp1*	1427097_AT
predicted gene 13422; protein tyrosine phosphatase 4a2	Ptp4a2	1460707_AT
predicted gene 5801; ubiquitin-conjugating enzyme E2, J2 homolog (yeast)	Ube2j2	1426138_A_AT
protein inhibitor of activated STAT 2	Pias2	1426456_A_AT
protein kinase N2	Pkn2	1437296_AT

protein kinase, AMP-activated, alpha 2 catalytic subunit	Prkaa2	1429463_AT<br>1429464_AT<br>1434766_AT
protein phosphatase 1G (formerly 2C), magnesium-dependent, gamma isoform	Ppm1g	1416792_AT
protein phosphatase 6, catalytic subunit	Ppp6c	1424346_AT
protein tyrosine phosphatase, non-receptor type 11	Ptpn11	1427699_A_AT
protein tyrosine phosphatase, receptor type, F	Ptprf	1420842_AT
protein tyrosine phosphatase, receptor type, K	Ptprk	1423277_AT
quiescin Q6 sulfhydryl oxidase 1	Qsox1*	1420832_AT
ribosomal protein S6 kinase, polypeptide 1	Rps6kb1	1460705_AT<br>1454956_AT
ring finger protein 2	Rnf2*	1451519_AT
serine/threonine kinase 24 (STE20 homolog, yeast)	Stk24	1426247_AT
seven in absentia 2	Siah2*	1448170_AT
similar to ubiquitin-conjugating enzyme UbcM2; ubiquitin-conjugating enzyme E2E 3, UBC4/5 homolog (yeast)	Ube2e3	1448670_AT
suppressor of zeste 12 homolog (Drosophila)	Suz12*	1452364_AT
testis specific protein kinase 1	Tesk1	1450662_AT
transformation/transcription domain-associated protein	Trrap*	1416532_AT
transglutaminase 2, C polypeptide	Tgm2*	1433428_X_AT<br>1417500_A_AT
ubiquitin specific peptidase 12; predicted gene 8902	Usp12	1434483_AT
ubiquitin specific peptidase 9, X chromosome	Usp9x	1450039_AT
ubiquitin-conjugating enzyme E2D 2; predicted gene 9762	Ube2d2	1416477_AT
ubiquitin-conjugating enzyme E2G 2	Ube2g2*	1417032_AT
ubiquitin-conjugating enzyme E2K (UBC1 homolog, yeast)	Ube2k	1417186_AT
v-raf-leukemia viral oncogene 1	Raf1	1425419_A_AT
**GO:0006467~protein thiol-disulfide exchange**		
ERO1-like (S. cerevisiae)	Ero1l*	1449324_AT
ERO1-like beta (S. cerevisiae)	Ero1lb*	1434714_AT<br>1425705_A_AT
quiescin Q6 sulfhydryl oxidase 1	Qsox1*	1420832_AT
**GO:0006413~translational initiation**		
eukaryotic translation initiation factor 1B	Eif1b	1428272_AT
eukaryotic translation initiation factor 3, subunit A	Eif3a	1416661_AT
eukaryotic translation initiation factor 3, subunit C; similar to Eukaryotic translation initiation factor 3, subunit 8	Eif3c	1415859_AT
hypothetical LOC630527; eukaryotic translation initiation factor 4E; similar to eukaryotic translation initiation factor 4E	Eif4e	1450908_AT
similar to Eif2s2 protein; eukaryotic translation initiation factor 2, subunit 2 (beta)	Eif2s2	1456617_A_AT<br>1417713_AT
similar to Eukaryotic translation initiation factor 5; eukaryotic translation initiation factor 5	Eif5	1454664_A_AT
**GO:0006520~cellular amino acid metabolic process**		
5,10-methylenetetrahydrofolate reductase	Mthfr	1434087_AT
RIKEN cDNA 3930401K13 gene	3930401<br>K13Rik	1451994_S_AT
RIKEN cDNA 4930402E16 gene	4930402<br>E16Rik	1459869_X_AT
alanyl-tRNA synthetase	Aars	1451083_S_AT<br>1423685_AT
glutamate dehydrogenase 1; predicted gene 5902	Glud1	1448253_AT
glutamate-cysteine ligase, catalytic subunit	Gclc*	1455959_S_AT<br>1424296_AT
glutamate-cysteine ligase, modifier subunit	Gclm	1418627_AT
glutamine fructose-6-phosphate transaminase 1	Gfpt1	1428715_AT
glycine decarboxylase	Gldc	1416049_AT
guanine monphosphate synthetase; predicted gene 7282	Gmps	1433567_AT
isoleucine-tRNA synthetase	Iars	1452154_AT<br>1426705_S_AT
leucyl-tRNA synthetase	Lars	1448403_AT
methionine-tRNA synthetase	Mars	1455951_AT

seryl-aminoacyl-tRNA synthetase	Sars	1452000_S_AT<br>1426257_A_AT
threonyl-tRNA synthetase	Tars	1460323_AT
**GO:0048008~platelet-derived growth factor receptor signaling pathway**		
RIKEN cDNA 6820431F20 gene	6820431<br>F20Rik	1436740_AT
myosin IE	Myo1e	1428509_AT
phosphatase and tensin homolog	Pten*	1455728_AT<br>1422553_AT
platelet-derived growth factor, C polypeptide	Pdgfc	1419123_A_AT
sphingosine phosphate lyase 1	Sgpl1	1415892_AT
**GO:0006479~protein amino acid methylation**		
B-cell translocation gene 1, anti-proliferative; similar to myocardial vascular inhibition factor	Btg1*	1426083_A_AT
G1 to S phase transition 1	Gspt1*	1452168_X_AT<br>1426736_AT
eukaryotic translation termination factor 1	Etf1*	1451208_AT
myeloid/lymphoid or mixed-lineage leukemia 5	Mll5*	1439108_AT
nuclear receptor-binding SET-domain protein 1	Nsd1*	1435088_AT
suppressor of zeste 12 homolog (Drosophila)	Suz12*	1452364_AT
**GO:0007435~salivary gland morphogenesis**		
cadherin 1	Cdh1	1448261_AT
dystroglycan 1	Dag1	1426778_AT<br>1426779_X_AT
neuropilin 1	Nrp1	1457198_AT
transglutaminase 2, C polypeptide	Tgm2*	1433428_X_AT<br>1417500_A_AT
twisted gastrulation homolog 1 (Drosophila)	Twsg1	1450388_S_AT
**GO:0051258~protein polymerization**		
fibrinogen alpha chain	Fga	1424279_AT
fibrinogen beta chain	Fgb	1428079_AT
fibrinogen gamma chain	Fgg	1416025_AT
tubulin, alpha 4A	Tuba4a	1417374_AT<br>1417373_A_AT
tubulin, beta 2c, psuedogene 1; tubulin, beta 2C; tubulin, beta 2c, pseudogene 2	Tubb2c	1423642_AT
tubulin, beta 5	Tubb5	1416256_A_AT
**GO:0016567~protein ubiquitination**		
Bmi1 polycomb ring finger oncogene	Bmi1*	1417493_AT
F-box protein 4	Fbxo4*	1427121_AT
G1 to S phase transition 1	Gspt1*	1452168_X_AT<br>1426736_AT
HECT, UBA and WWE domain containing 1	Huwe1*	1415703_AT
PEST proteolytic signal containing nuclear protein	Pcnp*	1452735_AT
predicted gene 13416; WW domain containing E3 ubiquitin protein ligase 1	Wwp1*	1427097_AT
ring finger protein 2	Rnf2*	1451519_AT
seven in absentia 2	Siah2*	1448170_AT
suppressor of zeste 12 homolog (Drosophila)	Suz12*	1452364_AT
**GO:0008380~RNA splicing**		
PRP40 pre-mRNA processing factor 40 homolog A (yeast)	Prpf40a*	1420917_AT
PRP6 pre-mRNA splicing factor 6 homolog (yeast)	Prpf6*	1424036_AT<br>1454789_X_AT
RIKEN cDNA 0610009D07 gene	0610009<br>D07Rik*	1417054_A_AT
RIKEN cDNA 3300001P08 gene	3300001<br>P08Rik*	1451485_AT
expressed sequence AU014645; nuclear cap binding protein subunit 1	Ncbp1*	1433663_S_AT
heterogeneous nuclear ribonucleoprotein K; predicted gene 7964	Hnrnpk*	1448176_A_AT
predicted gene 10232; predicted gene 6978; U2 small nuclear ribonucleoprotein auxiliary factor (U2AF) 1	U2af1*	1422509_AT

predicted gene 13886; TAR DNA binding protein	Tardbp*	1423723_S_AT
predicted gene 5446; similar to FUS interacting protein (serine-arginine rich) 1; FUS interacting protein (serine-arginine rich) 1	Fusip1*	1418527_A_AT
predicted gene 7509; splicing factor, arginine/serine-rich 3 (SRp20); similar to MGC89287 protein; predicted gene 9761; predicted gene 7083	Sfrs3*	1454993_A_AT
predicted gene 8696; predicted gene 5616; predicted gene 8948; NHP2 non-histone chromosome protein 2-like 1 (S. cerevisiae); predicted gene 13642; predicted gene 7042; similar to NHP2 non-histone chromosome protein 2-like 1; similar to Nhp2 non-histone chromosome protein 2-like 1; predicted gene 13311; similar to nuclear protein-NHP2-like protein; predicted gene 6637	Nhp2l1*	1416973_AT
serine/arginine repetitive matrix 2; similar to Serine/arginine repetitive matrix protein 2; similar to retinitis pigmentosa GTPase regulator	Srrm2*	1437638_AT
serine/threonine kinase receptor associated protein	Strap*	1416566_AT
similar to protein phosphatase 4, regulatory subunit 2; similar to Protein phosphatase 4, regulatory subunit 2; protein phosphatase 4, regulatory subunit 2	Ppp4r2*	1433850_AT
splicing factor proline/glutamine rich (polypyrimidine tract binding protein associated); similar to PTB-associated splicing factor	Sfpq*	1423795_AT<br>1438458_A_AT<br>1436898_AT
splicing factor, arginine/serine rich 9	Sfrs9*	1417727_AT
splicing factor, arginine/serine-rich 2 (SC-35)	Sfrs2*	1415807_S_AT
splicing factor, arginine/serine-rich 7	Sfrs7*	1424883_S_AT
synaptotagmin binding, cytoplasmic RNA interacting protein	Syncrip*	1422769_AT
transformer 2 beta homolog (Drosophila); predicted gene 6439	Tra2b*	1419543_A_AT
ubiquitin specific peptidase 39	Usp39*	1460209_AT<br>1437007_X_AT
**GO:0006397~mRNA processing**		
CCR4-NOT transcription complex, subunit 6-like	Cnot6l	1425481_AT
PRP40 pre-mRNA processing factor 40 homolog A (yeast)	Prpf40a*	1420917_AT
PRP6 pre-mRNA splicing factor 6 homolog (yeast)	Prpf6*	1454789_X_AT<br>1424036_AT
RIKEN cDNA 0610009D07 gene	0610009<br>D07Rik*	1417054_A_AT
RIKEN cDNA 3300001P08 gene	3300001<br>P08Rik*	1451485_AT
RNA binding motif protein 25	Rbm25	1425523_AT
apolipoprotein B mRNA editing enzyme, catalytic polypeptide 1	Apobec1	1451755_A_AT
cleavage and polyadenylation specific factor 2	Cpsf2	1420937_AT
expressed sequence AU014645; nuclear cap binding protein subunit 1	Ncbp1*	1433663_S_AT
heterogeneous nuclear ribonucleoprotein K; predicted gene 7964	Hnrnpk*	1448176_A_AT
heterogeneous nuclear ribonucleoprotein L-like; glutathione peroxidase 4	Hnrpll	1425255_S_AT
poly (A) polymerase alpha	Papola	1455836_AT
poly(A) binding protein, nuclear 1	Pabpn1	1422849_A_AT<br>1422848_A_AT
predicted gene 10232; predicted gene 6978; U2 small nuclear ribonucleoprotein auxiliary factor (U2AF) 1	U2af1*	1422509_AT
predicted gene 13886; TAR DNA binding protein	Tardbp*	1423723_S_AT
predicted gene 5446; similar to FUS interacting protein (serine-arginine rich) 1; FUS interacting protein (serine-arginine rich) 1	Fusip1*	1418527_A_AT
predicted gene 7509; splicing factor, arginine/serine-rich 3 (SRp20); similar to MGC89287 protein; predicted gene 9761; predicted gene 7083	Sfrs3*	1454993_A_AT
predicted gene 8696; predicted gene 5616; predicted gene 8948; NHP2 non-histone chromosome protein 2-like 1 (S. cerevisiae); predicted gene 13642; predicted gene 7042; similar	Nhp2l1*	1416973_AT

to NHP2 non-histone chromosome protein 2-like 1; similar to Nhp2 non-histone chromosome protein 2-like 1; predicted gene 13311; similar to nuclear protein-NHP2-like protein; predicted gene 6637		
serine/arginine repetitive matrix 2; similar to Serine/arginine repetitive matrix protein 2; similar to retinitis pigmentosa GTPase regulator	Srrm2*	1437638_AT
serine/threonine kinase receptor associated protein	Strap*	1416566_AT
similar to protein phosphatase 4, regulatory subunit 2; similar to Protein phosphatase 4, regulatory subunit 2; protein phosphatase 4, regulatory subunit 2	Ppp4r2*	1433850_AT
splicing factor proline/glutamine rich (polypyrimidine tract binding protein associated); similar to PTB-associated splicing factor	Sfpq*	1436898_AT<br>1438458_A_AT<br>1423795_AT
splicing factor, arginine/serine rich 9	Sfrs9*	1417727_AT
splicing factor, arginine/serine-rich 2 (SC-35)	Sfrs2*	1415807_S_AT
splicing factor, arginine/serine-rich 7	Sfrs7*	1424883_S_AT
stem-loop binding protein; predicted gene 8396	Slbp	1460168_AT
synaptotagmin binding, cytoplasmic RNA interacting protein	Syncrip*	1422769_AT
transformer 2 beta homolog (Drosophila); predicted gene 6439	Tra2b*	1419543_A_AT
ubiquitin specific peptidase 39	Usp39*	1460209_AT<br>1437007_X_AT
**GO:0016481~negative regulation of transcription**		
Bmi1 polycomb ring finger oncogene	Bmi1*	1417493_AT
YY1 associated factor 2	Yaf2	1423399_A_AT
ataxin 1	Atxn1*	1445695_AT<br>1438294_AT
bromodomain PHD finger transcription factor	Bptf*	1427311_AT<br>1427310_AT
frizzled homolog 1 (Drosophila)	Fzd1	1437284_AT
glutamate-cysteine ligase, catalytic subunit	Gclc*	1424296_AT<br>1455959_S_AT
inhibitor of DNA binding 2	Id2*	1453596_AT
metadherin	Mtdh*	1455129_AT
nuclear receptor-binding SET-domain protein 1	Nsd1*	1435088_AT
polycomb group ring finger 6	Pcgf6*	1424081_AT
predicted gene 13416; WW domain containing E3 ubiquitin protein ligase 1	Wwp1*	1427097_AT
purine rich element binding protein B	Purb	1428254_AT<br>1419642_AT
ring finger protein 2	Rnf2*	1451519_AT
serine/threonine kinase receptor associated protein	Strap*	1416566_AT
similar to Transcriptional repressor p66 alpha (GATA zinc finger domain-containing protein 2A); GATA zinc finger domain containing 2A	Gatad2a	1451197_S_AT<br>1423992_AT
similar to hCG45299; purine rich element binding protein A	Pura	1420628_AT<br>1456898_AT
ski sarcoma viral oncogene homolog (avian)	Ski*	1426373_AT<br>1429192_AT
suppressor of defective silencing 3 homolog (S. cerevisiae)	Suds3	1427896_AT
suppressor of zeste 12 homolog (Drosophila)	Suz12*	1452364_AT
transducin (beta)-like 1 X-linked	Tbl1x*	1434643_AT<br>1455042_AT<br>1434644_AT
transducin-like enhancer of split 1, homolog of Drosophila E(spl)	Tle1*	1422751_AT
tripartite motif-containing 27	Trim27*	1448101_S_AT<br>1438376_S_AT
zinc finger homeobox 3	Zfhx3*	1449947_S_AT
**GO:0006829~zinc ion transport**		
solute carrier family 30 (zinc transporter), member 4	Slc30a4	1418843_AT
solute carrier family 30, member 10	Slc30a1<br>0	1438751_AT
solute carrier family 39 (metal ion transporter), member 8	Slc39a8	1416832_AT

solute carrier family 39 (zinc transporter), member 1; similar to Zinc transporter ZIP1 (Zinc-iron regulated transporter-like) (Solute carrier family 39 member 1)	Slc39a1	1424424_AT
solute carrier family 39 (zinc transporter), member 7	Slc39a7	1416949_S_AT
**GO:0043161~proteasomal ubiquitin-dependent protein catabolic process**		
CD2-associated protein	Cd2ap	1420908_AT<br>1420906_AT
Der1-like domain family, member 2	Derl2	1448438_AT
ER degradation enhancer, mannosidase alpha-like 3	Edem3	1460401_AT
F-box protein 6	Fbxo6	1417501_AT
PEST proteolytic signal containing nuclear protein	Pcnp*	1452735_AT
transducin (beta)-like 1 X-linked	Tbl1x*	1455042_AT<br>1434643_AT<br>1434644_AT
ubiquitin-conjugating enzyme E2G 2	Ube2g2*	1417032_AT
**GO:0016570~histone modification**		
Bmi1 polycomb ring finger oncogene	Bmi1*	1417493_AT
CREB binding protein	Crebbp*	1436983_AT
HECT, UBA and WWE domain containing 1	Huwe1*	1415703_AT
histone deacetylase 2	Hdac2*	1445684_S_AT
myeloid/lymphoid or mixed-lineage leukemia 5	Mll5*	1439108_AT
nuclear receptor-binding SET-domain protein 1	Nsd1*	1435088_AT
ring finger protein 2	Rnf2*	1451519_AT
suppressor of zeste 12 homolog (Drosophila)	Suz12*	1452364_AT
transformation/transcription domain-associated protein	Trrap*	1416532_AT
**GO:0006357~regulation of transcription from RNA polymerase II promoter**		
Bmi1 polycomb ring finger oncogene	Bmi1*	1417493_AT
CREB binding protein	Crebbp*	1436983_AT
E74-like factor 1	Elf1	1417540_AT
E74-like factor 2	Elf2	1428045_A_AT
ELL associated factor 1	Eaf1	1433555_AT
MAD homolog 1 (Drosophila)	Smad1	1459843_S_AT
PRP6 pre-mRNA splicing factor 6 homolog (yeast)	Prpf6*	1424036_AT<br>1454789_X_AT
activating transcription factor 5	Atf5	1425927_A_AT
agrin	Agrn*	1426670_AT
aryl hydrocarbon receptor nuclear translocator-like	Arntl	1425099_A_AT
ataxin 1	Atxn1*	1445695_AT<br>1438294_AT
bromodomain PHD finger transcription factor	Bptf*	1427311_AT<br>1427310_AT
cAMP responsive element binding protein 1	Creb1*	1452901_AT
circadian locomoter output cycles kaput	Clock	1418660_AT<br>1418659_AT
core binding factor beta	Cbfb	1460716_A_AT
inhibitor of DNA binding 2	Id2*	1453596_AT
melanoma antigen, family D, 1	Maged1	1450062_A_AT
metadherin	Mtdh*	1455129_AT
nuclear factor of activated T-cells 5	Nfat5	1438999_A_AT
nuclear receptor subfamily 5, group A, member 2	Nr5a2(LRH-1)	1420410_AT
nuclear receptor-binding SET-domain protein 1	Nsd1*	1435088_AT
one cut domain, family member 1	Onecut1	1456974_AT
polycomb group ring finger 6	Pcgf6*	1424081_AT
predicted gene 12372; predicted gene 14335; similar to RNA polymerase II TATA box binding protein-associated factor G; TAF9 RNA polymerase II, TATA box binding protein (TBP)-associated factor	Taf9	1451509_AT
ring finger protein 2	Rnf2*	1451519_AT
serine/threonine kinase receptor associated protein	Strap*	1416566_AT
similar to MAD homolog 4 (Drosophila); MAD homolog 4 (Drosophila)	Smad4	1422485_AT
similar to Nuclear receptor coactivator 1 (NCoA-1) (Steroid	Ncoa1	1418594_A_AT

receptor coactivator 1) (SRC-1) (Nuclear receptor coactivator protein 1) (mNRC-1); nuclear receptor coactivator 1		
ski sarcoma viral oncogene homolog (avian)	Ski*	1426373_AT<br>1429192_AT
suppressor of zeste 12 homolog (Drosophila)	Suz12*	1452364_AT
thyroid hormone receptor associated protein 3; predicted gene 5898	Thrap3	1427408_A_AT
transducin (beta)-like 1 X-linked	Tbl1x*	1455042_AT<br>1434643_AT<br>1434644_AT
transducin-like enhancer of split 1, homolog of Drosophila E(spl)	Tle1*	1422751_AT
tripartite motif-containing 27	Trim27*	1438376_S_AT<br>1448101_S_AT
zinc finger homeobox 3	Zfhx3*	1449947_S_AT
zinc finger protein 148	Zfp148	1449069_AT<br>1427730_A_AT

* : Genes appearing more than two times in the functional groupings.
